# Supplementary figures and images for: When pits fill up: Supply and demand for safe pit-emptying services in Kisumu, Kenya
Source: PLoS One. 2020 Sep 3;15(9):e0238003. doi: 10.1371/journal.pone.0238003 (PMC7470379; doi:10.1371/journal.pone.0238003)

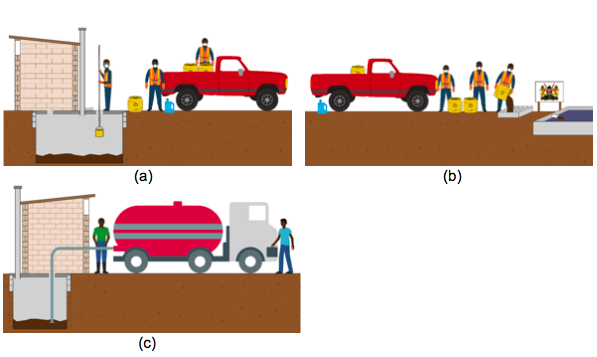

Supplement: S1 Fig — Graphics of (a) formal manual emptying, and (b) formal manual disposal, and (c) VTOs. We used to explain these services to study participants. (TIF) [file pone.0238003.s001.tif]

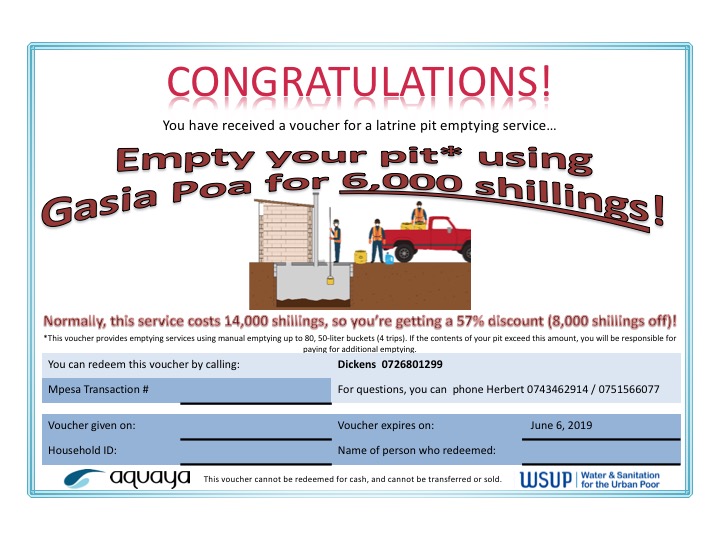

Supplement: S2 Fig — The randomized starting price points were: 3,000/6,000/8,000/11,000 KES (30/60/80/110 USD) for Gasia Poa emptying services. The full cost for Gasia Poa emptying services was 14,000 KES (140 USD). (TIF) [file pone.0238003.s002.tif]
