# Supplementary material for: When pits fill up: Supply and demand for safe pit-emptying services in Kisumu, Kenya
Source: PLoS One. 2020 Sep 3;15(9):e0238003. doi: 10.1371/journal.pone.0238003 (PMC7470379; doi:10.1371/journal.pone.0238003)
Supplement: S1 Table — (DOCX) [file pone.0238003.s003.docx]

**Table S1. Financial analysis of the capital expenditures (CAPEX) and operating expenditures (OPEX) for safe emptying in Kisumu, Kenya and comparisons with customer willingness-to-pay.**

|  | **No sewer expansion** | | **With sewer expansion** | | **Notes** |
| --- | --- | --- | --- | --- | --- |
|  | **KES** | **USD** | **KES** | **USD** |  |
| **Assumptions** | | | | | |
| Total population in Kisumu city | 419,072 | | | | Based on SFD^a^ |
| % of population currently lacking safe emptying & transport services | 62% | | | | Based on SFD^a^ |
| % of population to be served by upcoming sewer expansion | 0% | | 20% | | KIWASCO is planning to increase sewer coverage to 40% by 2022^b^, which represents a 20% expansion over the current coverage^c^ |
| Number of households in need of safe emptying & transport services | 51,965 | | 35,202 | | Assuming an average household size of 5 people (according to survey findings). This is 62% and 42% of the total population (419,072) divided by five people per households. |
| Number of households to be served by VTO services | 38,974 | | 26,402 | | Assuming a 75%-25% breakdown between VTO and formal manual emptying services, based on market assessment research^c,d^ |
| Number of households to be served by formalized manual emptiers | 12,991 | | 8,801 | |  |
| Average number of households per toilet | 6 | | | | From our survey data |
| Number of toilets in need of safe emptying & transport services | 8,661 | | 5,867 | | Dividing the number of households in need of each service by six households sharing a toilet |
| Number of toilets to be served by VTO services | 6,496 | | 4,400 | |  |
| Number of toilets to be served by formalized manual emptiers | 2,165 | | 1,467 | |  |
| **CAPEX** | | | | | |
| Number of new exhauster trucks | 8 | | 4 | | One exhauster truck can serve 3,200 households. The 9 existing trucks are currently operating at 50% capacity^c^; therefore, they can serve 3200 x 9 x 50% = 14,400 more people. We calculated the additional trucks from the remaining population (38,974 or 26,402) minus 14,400 divided by 3200. |
| Number of new pick-up trucks | 7 | | 4 | | One pick-up truck allows formal manual emptiers to serve 1,600 households. The 3 existing trucks are currently operating at 33% capacity^c^; therefore, they can serve 1600 x 3 x 67% = 3,216 more people. We calculated the additional trucks from the remaining population (12,991 or 8,801 or 26, 402) minus 3,216 divided by 1600. |
| Number of new tractors | 1 | | 1 | | One tractor will be needed to reach low-accessibility areas^c^. |
| Unit cost: exhauster truck | KES 8,000,000 | USD 80,000 | KES 8,000,000 | USD 80,000 | From market assessment |
| Unit cost pick-up truck | KES 2,500,000 | USD 25,000 | KES 2,500,000 | USD 25,000 | From market assessment |
| Unit cost: tractor | KES 2,500,000 | USD 25,000 | KES 2,500,000 | USD 25,000 | From market assessment |
| ***TOTAL CAPEX*** | **KES 84,000,000** | **USD 840,000** | **KES 44,500,000** | **USD 445,000** | Sum of number of trucks multiplied by unit costs |
| **OPEX** | | | | | |
| Average emptying price: VTO services | KES 5000 | USD 50 | KES 5000 | USD 50 | Prices ranged between 40 USD and 60 USD per emptying job. |
| Average emptying price: formal manual emptying services | KES 9500 | USD 95 | KES 9500 | USD 95 | Prices ranged between 70 USD and 120 USD per emptying job. |
| Estimated average toilet emptying frequency (months) | 12 | | | | Assumption based on survey data. Among voucher recipients (who all stated planning to empty their pit in the next three months), approximately half had their pit last emptied less than one year ago, and approximately half had it last emptied more than one year ago or never (Table 2). We therefore used one year as a plausible average frequency of emptying in Kisumu. |
| Annual cost per toilet served: VTO services | KES 5,000 | USD 50 | KES 5,000 | USD 50 | We assume that OPEX is entirely captured in the consumer price, i.e. that the consumer price covers all operational expenses such as fuel, labor, and asset depreciation. These are the average emptying price (50 or 95 USD) multiplied by 1 emptying per year. |
| Annual cost per toilet served: formal manual emptying services | KES 9,500 | USD 95 | KES 9,500 | USD 95 |  |
| **TOTAL Annual OPEX** | **KES 53,000,000** | **USD 530,000** | **KES 35,900,000** | **USD 359,000** | We multiplied the annual cost per toilet for each emptying service by the number of toilets allocated to each service. |
| **WTP** | | | | | |
| Price that 80% of financially responsible household are WTP per emptying job: VTO services | KES 1,200 | USD 12 | KES 1,200 | USD 12 | From our survey data. |
| Price that 80% of financially responsible household are WTP per emptying job: formal manual emptying services | KES 2,100 | USD 21 | KES 2,100 | USD 21 | From our survey data. |
| Estimated average toilet emptying frequency (months) | 12 | | | | Assumption based on survey data (see justification above). |
| Annual WTP per toilet served: VTO services | KES 1,200 | USD 12 | KES 1,200 | USD 12 | This is the price that 80% are WTP (12 or 21 USD) multiplied by 1 emptying per year. |
| Annual WTP per toilet served: formal manual emptying services | KES 2,100 | USD 21 | KES 2,100 | USD 21 |  |
| **TOTAL Annual WTP** | **KES 12,300,000** | **USD 123,000** | **KES 8,400,000** | **USD**  **84,000** | We multiplied the annual WTP per toilet for each emptying service by the number of toilets allocated to each service |
| **Gap between annual OPEX and annual WTP** | **KES 40,700,000** | **USD 407,000** | **KES 27,600,000** | **USD 276,000** | This is the difference between the annual operating expenses (OPEX) and annual WTP |

^a^ Furlong, C., & Jooust, L. (2016). *SFD Kisumu*. Kenya.

^b^ KIWASCO. Key informant interview. August 19, 2019.

^c^ Market assessment: key informant interviews. 2019.

^d^ Peletz, R., Feng, A., MacLeod, C., Vernon, D., Wang, T., Kones, J., Delaire, C., Haji, S., Khush, R., n.d. Expanding safe fecal sludge management in Kisumu, Kenya: a comparison of incentivized latrine pit emptying service model interventions. *Forthcoming*.
