# Supplementary material for: When pits fill up: Supply and demand for safe pit-emptying services in Kisumu, Kenya
Source: PLoS One. 2020 Sep 3;15(9):e0238003. doi: 10.1371/journal.pone.0238003 (PMC7470379; doi:10.1371/journal.pone.0238003)
Supplement: S2 Table — (DOCX) [file pone.0238003.s004.docx]

**Table S2. Bivariate subgroup analysis of stated WTP.**

|  | **Gasia Poa** | | | | **VTOs** | | | |
| --- | --- | --- | --- | --- | --- | --- | --- | --- |
| **Subgroup** | **N** | **Mean WTP (KES)** | **Median WTP (KES)** | **P-value (t-test of means)** | **N** | **Mean WTP (KES)** | **Median WTP (KES)** | **P-value (t-test of means)** |
| **Gender**  Female  Male | 450  344 | 3,098  3,883 | 3,000  3,000 | <0.01 | 450  345 | 2,045  2,361 | 2,000  2,000 | <0.01 |
| **Wealth Quintile**  Lowest  Low  Middle  High  Highest | 143  152  160  167  172 | 3,352  3,188  3,274  3,489  3,834 | 3,000  3,000  3,000  3,000  3,000 | 0.048 | 143  152  160  167  173 | 1,881  2,074  2,209  2,339  2,351 | 1,500  2,000  2,000  2,500  2,500 | 0.015 |
| **Home Owner**  No  Yes | 92  702 | 3,614  3,415 | 3,000  3,000 | 0.39 | 93  702 | 2,602  2,127 | 3,000  2,000 | <0.01 |
| **Education**  None  Primary  Secondary  Post-secondary | 57  314  251  169 | 2,089  3,245  3,601  3,988 | 2,000  3,000  3,000  3,000 | <0.01 | 57  314  250  171 | 1,788  2,043  2,176  2,570 | 2,000  2,000  2,000  2,500 | <0.01 |
| **Age**  <35  35-49  ≥50+ | 249  262  281 | 3,869  3,514  2,971 | 3,000  3,000  3,000 | <0.01 | 249  262  281 | 2,441  2,294  1,844 | 2,250  2,000  2,000 | <0.01 |
| **Married**  Not married  Married | 283  511 | 3,284  3,523 | 3,000  3,000 | 0.13 | 284  511 | 1,999  2,284 | 2,000  2,000 | 0.01 |
| **Years lived in compound**  0-9  10-29  ≥30 | 194  334  263 | 3,608  3,407  3,345 | 3,000  3,000  3,000 | 0.40 | 194  335  263 | 2,517  2,209  1,895 | 2,500  2,000  2,000 | <0.01 |
| **Reported sharing latrine**  No  Yes | 127  667 | 3,092  3,504 | 3,000  3,000 | 0.04 | 127  558 | 2,032  2,211 | 2,000  2,000 | 0.18 |
| **Have M-Pesa**  No  Yes | 31  763 | 1,968  3,498 | 2,000  3,000 | <0.01 | 31  764 | 1,500  2,210 | 1,000  2,000 | <0.01 |
| **Have children <5 years**  None  Kids <5 | 484  310 | 3,487  3,361 | 3,000  3,000 | 0.41 | 485  310 | 2,148  2,235 | 2,000  2,000 | 0.39 |
| **Toilet located < 50 meters from a road**  Yes  No | 600  194 | 3,445  3,417 | 3,000  3,000 | 0.88 | 601  194 | 2,385  1,554 | 2,500  1,000 | <0.01 |
| **Big expense in past 3 months^a^**  No  Yes | 46  73 | 3,413  3,771 | 3,000  3,000 | 0.39 | 46  73 | 2,024  2,345 | 2,000  2,500 | 0.23 |
| **Total population** | 794 | 3,438 | 3,000 |  | 795 | 2,182 | 2,000 |  |

^a^ Only asked for households that were followed up. Big expenditures most commonly included school fees (43% of households, median 25,000 KES) and medical expenses (15% of households, median 9500 KES).
