# Supplementary material for: When pits fill up: Supply and demand for safe pit-emptying services in Kisumu, Kenya
Source: PLoS One. 2020 Sep 3;15(9):e0238003. doi: 10.1371/journal.pone.0238003 (PMC7470379; doi:10.1371/journal.pone.0238003)
Supplement: S3 Table — (DOCX) [file pone.0238003.s005.docx]

**Table S3. Multivariate subgroup analysis of stated WTP.**

|  | **Gasia Poa** | | | **VTO** | | |
| --- | --- | --- | --- | --- | --- | --- |
| **Subgroup** | **Coeff** | **z** | **p-value** | **Coeff** | **z** | **p-value** |
| **Male** | 1.29 | 102.51 | <0.01 | 1.15 | 9.68 | 0.03 |
| **Age** | 0.99 | 1.00 | <0.01 | 0.99 | 0.06 | <0.01 |
| **Wealth Quintile** | 1.03 | 4.39 | 0.14 | 1.07 | 27.94 | <0.01 |
| **Education** | 1.08 | 8.76 | 0.03 | 1.00 | 0.88 | 0.90 |
| **Shared latrine** | 1.19 | 9.12 | 0.03 |  |  |  |
| **Home Owner** |  |  |  | 0.81 | 0.07 | <0.01 |
| **Married** |  |  |  | 0.99 | 0.78 | 0.8 |
| **Yeas lived in compound** |  |  |  | 1.00 | 0.43 | 0.40 |
