# Supplementary material for: When pits fill up: Supply and demand for safe pit-emptying services in Kisumu, Kenya
Source: PLoS One. 2020 Sep 3;15(9):e0238003. doi: 10.1371/journal.pone.0238003 (PMC7470379; doi:10.1371/journal.pone.0238003)
Supplement: S4 Table — (DOCX) [file pone.0238003.s006.docx]

**Table S4. Bivariate and multivariate subgroup analysis of voucher redemption.**

|  | **Bivariate** | | | **Multivariate** | | |
| --- | --- | --- | --- | --- | --- | --- |
| **Subgroup** | **Odds Ratio** | **95% CI** | **p-value** | **Odds Ratio** | **95% CI** | **p-value** |
| **Voucher discount (each 100 KES)** | 0.96 | 0.95-0.97 | <0.01 | 0.96 | 0.95-0.97 | <0.01 |
| **Wealth Quintile** | 1.35 | 1.16-1.57 | <0.01 | 1.23 | 1.05-1.44 | <0.01 |
| **VTO voucher** | 3.09 | 2.03-4.72 | <0.01 | 0.67 | 0.38-1.18 | 0.17 |
| **Shared latrine** | 0.47 | 0.23-0.80 | <0.01 | 0.83 | 0.46-1.52 | 0.56 |
| **Married** | 1.51 | 0.97-2.35 | 0.07 | 1.23 | 0.76-2.00 | 0.84 |
| **Secondary Education** | 1.42 | 0.95-2.23 | 0.09 | 1.13 | 0.72-1.78 | 0.6 |
| **Male** | 1.11 | 0.74-1.65 | 0.61 |  |  |  |
| **Home Owner** | 1.63 | 0.79-3.38 | 0.19 |  |  |  |
| **Under 40** | 1.19 | 0.79-1.78 | 0.40 |  |  |  |
| **Lived ≥20 years in compound** | 0.89 | 0.60-1.33 | 0.58 |  |  |  |
| **Have M-Pesa** | 2.67 | 0.62-11.5 | 0.19 |  |  |  |
| **Have children <5 years** | 0.93 | 0.62-1.40 | 0.73 |  |  |  |
| **Big expense in past 3 months^a^** | 0.97 | 0.47-2.0 | 0.94 |  |  |  |

^a^ Only asked for households that participated in the follow-up survey. Big expenditures most commonly included school fees (43% of households, median 25,000 KES) and medical expenses (15% of households, median 9500 KES).
