# Supplementary material for: When pits fill up: Supply and demand for safe pit-emptying services in Kisumu, Kenya
Source: PLoS One. 2020 Sep 3;15(9):e0238003. doi: 10.1371/journal.pone.0238003 (PMC7470379; doi:10.1371/journal.pone.0238003)
Supplement: S1 Text — (DOCX) [file pone.0238003.s007.docx]

# **Household Survey: Pre-Screening**

**[INSTRUCTIONS TO THE INTERVIEWER: The following statement should be read to every potential interviewee.]**

May I have a minute of your time, please? My name is ______________. I am a staff member at [ORGANIZATION] based in [COUNTRY]. I would like to invite you as a head of household to participate in our research study. The purpose of our research is to understand the ability of households to pay for sanitation options in [CITY] and will be conducted over 5 months. You are being asked to participate in this study because you live in one of the areas selected for the present study.

Is this a good time to talk?

Would you be willing to answer a few questions about yourself to find out if you might qualify for the study? You can stop at any time.

Thank you for your interest in our research study. May I begin?

**Pre-Interview Screening Questions –REQUIRED TO BE OVER AGE 18, LIVING IN THE COMPOUND, AND ONE OF THE HOUSEHOLD HEADS.**

1. Do you live on this compound?
2. Are you over 18 years old?
3. Do you take part in the household’s financial decisions?

**If subject qualifies (answers “Yes” to pre-screening), then continue to CONSENT below.** If not, thank them again for their time and interest.

IF RESPONDENT CONSENTS BUT IS UNABLE TO SPEND TIME, ASK: Is there a more convenient time when I could ask you some questions? **RECORD IN COMMCARE.**

# **Household Survey: Consent for Interview**

The interview will involve questions about living conditions in [CITY], access to and satisfaction with sanitation services. The interview should last no longer than 1 hour or until you feel that you have told me everything you want me to know. If you agree to participate in this research, I will conduct an interview with you now.

There are no right or wrong answers, so please be honest and tell us what is true for you. Information from this study may help increase understanding and awareness of what it is like to live in [CITY]. There are no personal risks or benefits to your participation. Everything that you say will be confidential, and we will not use your real name or any identifying information in any of our reports or papers. Some staff of the Aquaya Institute may sometimes look at your record for research purposes. The results will be used to inform government actors and companies about the ability to pay for existing sanitation costs.

**Your participation in this research is completely voluntary.** You can decline to answer any questions, and if you do not wish to continue, you can withdraw from the study at any time for any reason. You will not receive any monetary payment for this questionnaire. An alternative is to not participate in this study.

If you have any questions or concerns about the research, please feel free to contact me. I can be reached at [phone number] or [email address].

## If you agree to voluntarily participate in this study, please say so.

[ALL QUESTIONNAIRES WILL BE SAVED BY THE INTERVIEWER REGARDLESS OF THE RESPONDENT’S DECISION TO PARTICIPATE OR NOT TO PARTICIPATE.]

_____________________ _____________________ ___________________

Participant Name Participant Signature Date

_____________________ _____________________ ___________________

Interviewer Name Interviewer Signature Date

This research has been reviewed and approved by the [AMREF] Institutional Review Board. If you have any questions about your rights as a research participant, you can contact their office: [PHONE], [EMAIL], or [ADDRESS].

**Household Survey: CommCare**

| **Question** | **Answer** | **Question** | **Answer** | |
| --- | --- | --- | --- | --- |
| Date |  | Time Interview Began |  | |
| A_01  Interviewer Name | Enumerator 1­­­­­­­­­­­­­­__1  Enumerator 2 ___2  Enumerator 3___3  Enumerator 4_____4  Enumerator 5_____5  Supervisor ____6  Others______7 | Time Interview Ended |  | |
| A_01b  What number is this compound of those you have visited today? | | 1st compound of the day  2nd compound of the day  3rd compound of the day  4th compound of the day  5th compound of the day  6th compound of the day  7th compound of the day  8th compound of the day  9th compound of the day | | 1  2  3  4  5  6  7  8  9 |

| **Q No.** | **Pre-interview screening** | **Options/Answers** | |  | | **Skip to** |
| --- | --- | --- | --- | --- | --- | --- |
| A_11 | Take GPS measurement | | Lat: ________________. Long:________________. | | | |
| A_02 | Is a household head available to be interviewed who lives here and is ≥18 years old? | Yes  No | | 1  0 | | >>A_04 |
| A_03 | READ CONSENT FORM  Are you willing to participate in the study? | Yes, available now  Yes, though at a later time  No | | 1  2  0 | | >>A_06  >>End |
| A_04 | How many times have you visited this compound? | _______ | |  | | >>End if >2 |
| A_05 | May a household head who lives here and is ≥18 years old be available at a later time? | Yes  No | | 1  0 | | >>End |
| A_06 | When would be a good time to return? | __________________ | |  | | >>Return |
| A_07 | Name of LIA | Bandani  Manyatta A  Manyatta B  Nyabera  Nyalenda A  Nyalenda B  Nyamasaria  Obunga  Otonglo | | 1  2  3  4  5  6  7  8  9 | |  |
| A_08 | Name of respondent | |  | | | |
| A_09 | Phone number of respondent | |  | | | |
| A_10 | Do you know if this respondent is a tenant or a landlord? | Yes, landlord/homeowner  Yes, tenant  Don’t know | | 1  2  3 | |  |
| A_12 | Compound number | | __ __ __ __ __ __ . __  Enumerator #-Month-Day-Number.Owner Status | |  |  |
| IneligibleB | If the household was not eligible, why were they ineligible? | | No one was home after 3 tries.  Household was not willing to participate.  Household head was not over 18 years old.  Not a household – business, school, church, etc.  Landlord is not available at this time  Landlord does not live in this compound  They do not have access to a toilet in this compound  They only have access to a sewered toilet  Household not accessible  Other: ________ | | 1  2  3  4  5  6  7  8  9  96 |  |

| **Q No.** | **Demographics** | **Options/Answers** |  | **Skip to** |
| --- | --- | --- | --- | --- |
| B_01 | Does the respondent know their birthdate? | Yes  No | 1  0 | >>B_03 |
| B_02 | What is the birthdate of the respondent?  *If they don’t know their birth month, keep the day of the survey (i.e., February 12) and change the year.* | __ __ / __ __ / __ __ __ __  DAY / MONTH / YEAR |  | >>B_04 |
| B_03 | What is the approximate age of the respondent? | Years: | |  |
| B_04 | What is the gender of the respondent? | Male  Female | 1  2 |  |
| B_05 | Education level completed by respondent | None  Pre-primary  Primary  Post-primary training (e.g. vocational, tailoring, masonry)  Secondary  Mid-level college  University  Post-graduate  Other: _______________  Refuse to answer  Don’t know | 0  1  2  3  4  5  6  7  96  98  99 |  |
| B_06 | What is your marital status? | Married/In Union  Widowed  Separated/Divorced  Single  Other: _______________  Refuse to answer | 1  2  3  4  96  98 |  |
| B_07 | How many people are in your household?  *(Number of people who eat and sleep here more than 50% of the time in the last 12 months)* | Adults (18+ years, including respondent) |  |  |
| B_08 |  | Children 5-<18 years |  |  |
| B_09 |  | Children 0-4 years |  |  |
| B_10 | Does anyone in your household have difficulty walking or moving around? (e.g., elderly, persons with disability) | Yes  No  Refuse to answer | 1  0  98 |  |
| B_11 | How many years have you lived in this compound? | Lived here <1 year  Lived here ≥1 year: LIST YEARS___  Don’t know | 1  2  99 |  |
| B_12 | Do you own your dwelling?  *[If caretaker, asks if they pay rent or not.]* | Yes, own  No, rent  Housed for free  Other: _______________ | 1  0  2  96 |  |
| B_13 | Does anyone live in this compound other than your household? | Yes  No | 1  0 | >> B_20 |
| B_14 | Other than your household, how many households live in this compound (including the landlord if any)? | Households |  |  |
|  |  |  |  |  |
| B_16 | *[If B_12 = 1]*  Do you own any of these other houses? | Yes  No | 1  0 | >> C_01 |
| B_17 | How many of these other houses do you own? | Households | __ |  |
| B_18 | On average, what is the duration of rent for your tenants? | Less than 1 year  1-3 years  More than 3 years  Don’t know | 1  2  3  99 |  |
| B_19 | How much rent do you collect every month from all your tenants in this compound? | Amount KES_______  Refuse to answer  Don’t know | 98  99 |  |
| B_20 | *[If B_12=0 or 2]:*  Who owns this compound? | Private landlord  Government, county, railways  Church or community organization  Other:__________  Don’t know | 1  2  3  96  99 |  |
| B_21 | *[If B_12=0]:*  How much rent does your household pay every month?  *(99 for don’t know or refuse to answer)* | Amount KES_________  Refuse to answer  Don’t know | 98  99 |  |

| **Q No.** | **Sanitation Facilities**  ***(Establish Factors B and C)*** | **Options/Answers** | |  | **Skip to** |
| --- | --- | --- | --- | --- | --- |
| C_01 | *FOR TENANTS:*  Do you have access to a toilet in this compound? | Yes  No  Sometimes | | 1  0  2 | >>D_01 |
| C_02 | *FOR LANDLORDS/HOMEOWNERS:*  Do you own a toilet in this compound? | Yes  No | | 1  0 | >>D_01 |
| C_03 | Do you share this toilet facility with other households? | Yes  No | | 1  0 | >>C_05 |
| C_04 | Including your own household, how many households use this toilet facility? | Number of households | | __ |  |
| C_05 | May I see this toilet? | Yes  No | | 1  0 |  |
| C_06 | What kind of toilet facility is it?  [IF PERMISSION TO OBSERVE WAS NOT GRANTED, ASK RESPONDENT TO DESCRIBE, SHOW PHOTOS]. | **Flush or Pour Flush Toilet to**  Piped Sewer System  Septic Tank  Pit Latrine  Somewhere Else  Don't Know Where  **Pit Latrine**  Ventilated Improved Pit Latrine  Ventilated Improved, with Seat  Pit Latrine with Slab (Concrete, Plastic)  Pit Latrine with Slab (Mud, Wood)  Pit Latrine with Sitting Toilet  Pit Latrine Without Slab/Open Pit  **Other**  Composting Toilet (EcoSan)  Container-based Toilet  Bucket Toilet  Hanging Toilet/Hanging Latrine  No Facility/Bush/Field  Other: _______________ | | 1  2  3  4  5  6  14  7  15  8  9  10  11  12  13  0  96 |  |
| C_08 | How many squat holes (“doors”) does the toilet facility have? | _____________ squat holes | |  |  |
| *QUESTIONS C_10 THROUGH C_21 ARE OBSERVATIONS* | | | | | |
| C_09 | Did you observe this toilet during a previous survey? | Yes  No | | 1  0 | >>C_23 |
| C_10 | What is the material of the toilet/latrine walls?  *Covers more than 50% of the wall.* | Reed/grass  Mud/earthen  Mix of cement and mud  Brick  Wood  Sheet metal  Cement block  Plastic  No walls  Other: ________________  Cannot observe/don’t know | | 1  2  3  3  4  5  6  7  0  96  99 |  |
| C_10c | Is the superstructure stable enough for emptying by Gasia Poa? | Yes  No | | 1  0 |  |
| C_11 | What is the material of the toilet/latrine roof? | Reed/grass  Brick/clay tile  Wood  Sheet metal  Plastic  No roof  Other: ________________  Cannot observe/don’t know | | 1  2  3  4  5  0  96  99 |  |
| C_12 | What is the material of the toilet/latrine floor? | Mud/earthen  Brick  Wood  Cement  Ceramic pan + concrete  No flooring / open pit  Other: ________________  Cannot observe/don’t know | | 1  2  3  4  5  0  96  99 |  |
| C_13 | What is the condition of the toilet/latrine floor? | Spotless  Pretty clean, minimal dirt  Visible dirt or other trash  Filthy – covered in dirt/trash/feces  Cannot observe/don’t know | | 1  2  3  4  99 |  |
| C_14 | Is the toilet/latrine door lockable? | Yes, from the inside only  Yes, from the outside only  Yes, from both the inside and outside  No  No door  Cannot observe/don't know | | 1  2  3  0  4  99 | >>C_15  >>C_15  >>C_15 |
| C_14a | What type of lock does the door have on the inside? | String  Metal wire  Metal slide lock  Key lock  Other: ________________ | | 1  2  3  4  96 |  |
| C_14c | What type of lock does the door have on the outside? | String  Metal wire  Metal slide lock  Key lock  Other: ________________ | | 1  2  3  4  96 |  |
| C_15 | Is there a waste receptacle in or near the toilet/latrine? | Yes  No  Cannot observe/don’t know | | 1  0  99 |  |
| C_16 | Is there a handwashing facility in the vicinity of the toilet/latrine (less than 10 meters)? | Yes  No  Cannot observe/don’t know | | 1  0  99 | >>C_18  >>C_18 |
| C_17a | Is there water at the handwashing facility? | Yes  No  Cannot observe/don’t know | | 1  0  99 |  |
| C_17b | Is there soap at the handwashing facility? | Yes  No  Cannot observe/don’t know | | 1  0  99 |  |
| C_18 | Does the toilet have the following features? | Lighting  Seat  Handrail  Steps | | Y/N  Y/N  Y/N  Y/N |  |
| C_19 | *IF TOILET NOT CONNECTED TO SEWER:*  Is it possible to empty the pit without breaking the slab (hole > 30 cm)? | Yes  No | | 1  0 | >>C_20 |
| C_19b | *We can see that your slab needs to be broken for your pit to be emptied by Gasia Poa. Upon breaking your slab, you have two options. You can either convert the squat hole into a pour flush system by putting a ceramic pan or you can repair the slab. This is an additional 900-1300 KES for pour flush, or 500 KES to repair the concrete slab. This is an additional cost that is borne by the household. When we ask you the following WTP questions, the amounts do not include this additional cost. If it was time to empty your pit, would you be okay to have the slab broken in order to empty the pit?* | Yes  No | | 1  0 |  |
| C_20 | *IF TOILET NOT CONNECTED TO SEWER:*  Is the slab stable enough for emptying by Gasia Poa? | Yes  No  Cannot observe/don’t know | | 1  0  99 |  |
| C_21 | *IF TOILET NOT CONNECTED TO SEWER:*  Is the toilet accessible to VTOs (within 50m of road)? | Yes  No  Cannot observe/don’t know | | 1  0  99 |  |
| *END OF OBSERVATION* | | | | | |
| C_22 | *CALCULATE SANITATION QUALITY* | *If toilet is in the compound, improved, and with 3 max households per squat hole* | | HIGH |  |
|  |  | *Else or no toilet* | | LOW |  |
| C_23 | Who paid for the toilet to be constructed?  Select all that apply. | Me / my family  Landlord  Compound neighbors (how many): _____  Other: _________________  Don’t know | | 1  2  3  96  99 |  |
| C_24 | *If* C_23*=1:*  Where did you/your household get money to construct the toilet?  Select all that apply. | My own savings  Borrowed from family/friends  On credit  Other: __________________  Don’t know | | 1  2  3  96  99 |  |
| C_25 | *If* C_23*=1:*  How long did it take you/your household to finish the payments? | No time - paid immediately  Under one month  1-6 months  Over 6 months  Other: __________________  Don’t know | | 1  2  3  4  96  99 |  |
| C_26 | Are there any members of this household that usually don’t use this toilet facility? | Yes  No  Don’t know | | 1  0  99 |  |
| C_27 | On a scale of 1 – 5, how satisfied/unsatisfied are you with this toilet? [USE GRAPHIC SCALE] | Very unsatisfied  Somewhat unsatisfied  Average  Somewhat satisfied  Very satisfied  Don’t know | | 1  2  3  4  5  99 | >>C_29 |
| C_28 | What do you dislike about this toilet? Anything else?  *(probe as needed; select all that apply)* | Don’t like the type of toilet  Dirty  Bad smell  Health risk  Uncomfortable  Not suitable for children or elderly  Risk of falling  No privacy  Inconveniently located  Security concerns  No water  No receptacle for sanitary pads  Conflict with neighbors  Pit is full  Pit floods/overflows during rainy season  Blockages  Superstructure is too small  Uses too much water  Other: _______  Don’t know | | Y/N  Y/N  Y/N  Y/N  Y/N  Y/N  Y/N  Y/N  Y/N  Y/N  Y/N  Y/N  Y/N  Y/N  Y/N  Y/N  Y/N  Y/N  96  99 |  |
| *FOR LANDLORDS/HOMEOWNERS: if first toilet doesn’t serve all households he is responsible for*  *[If C_04 < Number of tenants in compound(B_14) +1 OR C_26=0]:* | | | | | |
| C_29 | Do you own another toilet facility in this compound? | Yes  No | | 1  0 | >>C_54 |
| C_30 | How many households use this toilet facility (including your household if applicable)? | Households | | ___ |  |
| C_31 | May I see this toilet? | Yes  No | | 1  0 |  |
| C_32 | What kind of toilet facility is it?  [IF PERMISSION TO OBSERVE WAS NOT GRANTED, ASK RESPONDENT TO DESCRIBE, SHOW PHOTOS]. | **Flush or Pour Flush Toilet to**  Piped Sewer System  Septic Tank  Pit Latrine  Somewhere Else  Don't Know Where  **Pit Latrine**  Ventilated Improved Pit Latrine  Pit Latrine with Slab  Pit Latrine with Sitting Toilet  Pit Latrine Without Slab/Open Pit  **Other**  Composting Toilet (EcoSan)  Container-based Toilet  Other: _______________ | | 1  2  3  4  5  6  7  8  9  10  11  96 |  |
| C_33 | How many squat holes does (“doors”) the toilet facility have? | _____________ squat holes | |  |  |
| *QUESTIONS C_35 THROUGH C_46 ARE OBSERVATIONS* | | | | | |
| C_34 | Did you observe this toilet during a previous survey? | Yes  No | | 1  0 | >>C_54 |
| C_35 | What is the material of the toilet/latrine walls?  *Covers more than 50% of the wall.* | Reed/grass  Mud/earthen  Mix of cement and mud  Brick  Wood  Sheet metal  Cement block  Plastic  No walls  Other: ________________  Cannot observe/don’t know | | 1  2  3  3  4  5  6  7  0  96  99 |  |
| C_35c | Is the superstructure stable enough for emptying by Gasia Poa? | Yes  No | | 1  0 |  |
| C_36 | What is the material of the toilet/latrine roof? | Reed/grass  Brick/clay tile  Wood  Sheet metal  Plastic  No roof  Other: ________________  Cannot observe/don’t know | | 1  2  3  4  5  0  96  99 |  |
| C_37 | What is the material of the toilet/latrine floor? | Mud/earthen  Brick  Wood  Cement  No flooring / open pit  Other: ________________  Cannot observe/don’t know | | 1  2  3  4  0  96  99 |  |
| C_38 | What is the condition of the toilet/latrine floor? | Spotless  Pretty clean, minimal dirt  Visible dirt or other trash  Filthy – covered in dirt/trash/feces  Unbearably dirty – I would not use  Cannot observe/don’t know | | 1  2  3  4  5  99 |  |
| C_39 | Is the toilet/latrine door lockable? | Yes, from the inside only  Yes, from the outside only  Yes, from both the inside and outside  No  No door  Cannot observe/don't know | | 1  2  3  0  4  99 | >C_39c  >>C_40  >>C_40  >>C_40 |
| C_39a | What type of lock does the door have on the inside? | String  Metal wire  Metal slide lock  Key lock  Other: ________________ | | 1  2  3  4  96 |  |
| C_39c | What type of lock does the door have on the outside? | String  Metal wire  Metal slide lock  Key lock  Other: ________________ | | 1  2  3  4  96 |  |
| C_40 | Is there a waste receptacle in or near the toilet/latrine? | Yes  No  Cannot observe/don’t know | | 1  0  99 |  |
| C_41 | Is there a handwashing facility in the vicinity of the toilet/latrine (less than 10 meters)? | Yes  No  Cannot observe/don’t know | | 1  0  99 | >>C_43  >>C_43 |
| C_42a | Is there water at the handwashing facility? | Yes  No  Cannot observe/don’t know | | 1  0  99 |  |
| C_42b | Is there soap at the handwashing facility? | Yes  No  Cannot observe/don’t know | | 1  0  99 |  |
| C_43 | Does the toilet/latrine have the following features? | Lighting  Seat  Handrail  Steps  Other: _________________  None | | Y/N  Y/N  Y/N  Y/N  96  0 |  |
| C_44 | *IF TOILET NOT CONNECTED TO SEWER:*  Is it possible to empty the pit without breaking the slab (hole > 30 cm)? | Yes  No | | 1  0 | >>C_45 |
| C_44b | *We can see that your slab needs to be broken for your pit to be emptied by Gasia Poa. Upon breaking your slab, you have two options. You can either convert the squat hole into a pour flush system by putting a ceramic pan or you can repair the slab. This is an additional 900-1300 KES for pour flush, or 500 KES to repair the concrete slab. This is an additional cost that is borne by the household. When we ask you the following WTP questions, the amounts do not include this additional cost. If it was time to empty your pit, would you be okay to have the slab broken in order to empty the pit?* | Yes  No | | 1  0 |  |
| C_45 | *IF TOILET NOT CONNECTED TO SEWER:*  Is the slab stable enough for emptying by Gasia Poa? | Yes  No  Cannot observe/don’t know | | 1  0  99 |  |
| C_46 | *IF TOILET NOT CONNECTED TO SEWER:*  Is the toilet accessible to VTOs (within 50m of road)? | Yes  No  Cannot observe/don’t know | | 1  0  99 |  |
| *END OF OBSERVATION* | | | | | |
| C_47 | *CALCULATE SANITATION QUALITY OF TOILET 2* | *If toilet is in the compound, improved, and with 3max households per compartment* | | HIGH |  |
|  |  | *Else or no toilet* | | LOW |  |
| C_48 | Who paid for the toilet to be constructed? (CAN CIRCLE MULTIPLE) | Me / my family  Compound neighbors (how many): _____  Other: _________________  Don’t know | | 1  2  96  99 |  |
| C_49 | *If* C_48*=1:*  Where did you/your household get money to construct the toilet? | My own savings  Borrowed from family/friends  On credit  Other: __________________ | | 1  2  3  4 |  |
| C_50 | *If* C_48*=1:*  How long did it take you/your household to finish the payments? | No time - paid immediately  Under one month  1-6 months  Over 6 months  Other: __________________ | | 1  2  3  4  5 |  |
| C_51 | Are there any members of this household that usually don’t use this toilet facility? | Yes  No  Don’t know | | 1  0  99 |  |
| C_52 | On a scale of 1 – 5, how satisfied/unsatisfied are you with this toilet? [USE GRAPHIC SCALE] | Very unsatisfied  Somewhat unsatisfied  Average  Somewhat satisfied  Very satisfied  Don’t know | | 1  2  3  4  5  99 | >>C_54 |
| C_53 | What do you dislike about this toilet? Anything else?  *(probe as needed; select all that apply)* | Don’t like the type of toilet  Dirty  Bad smell  Health risk  Uncomfortable  Not suitable for children or elderly  Risk of falling  No privacy  Inconveniently located  No water  Security concerns  No receptacle for sanitary pads  Conflict with neighbors  Pit is full  Pit floods/overflows during rainy season  Blockages  Superstructure is too small  Uses too much water  Other: _______  Don’t know | | Y/N  Y/N  Y/N  Y/N  Y/N  Y/N  Y/N  Y/N  Y/N  Y/N  Y/N  Y/N  Y/N  Y/N  Y/N  Y/N  Y/N  Y/N  96  99 |  |
| C_54 | *FOR TENANTS:*  *CALCULATE TENANT CATEGORY* | *If B_12 != 1 and C_22 = HIGH* | CAT 1 | |  |
|  |  | *If B_12 != 1 and C_22 = LOW* | CAT 2 | |  |
| C_55 | *FOR OWNERS:*  *CALCULATE OWNER CATEGORY* | *If B_12 =1 and C_02 = 0* | CAT 5 | |  |
|  |  | *If B_12 = 1 and C_22 = HIGH and C_47 = HIGH* | CAT 3 | |  |
|  |  | *If B_12 = 1 and (C_22 = LOW or C_47 = LOW)* | CAT 4 | |  |

| **Q No.** | **Remaining toilet questions** | **Options/Answers** |  | **Skip to** |
| --- | --- | --- | --- | --- |
| D_00 | ***If ANY of these scenarios are true****:*   - *C_01 = “0”* - *C_02 = “0”* - *B_12 != “1” and C_26 = “0”* - *B_12 = “1” and C_26 = “0” and C_29 = “0”* | | 1 |  |
|  | **Else:** | | 0 | >>E_01 |
| D_01 | What kind of toilet facility do members of your household usually use?  *(Those that do not use the first toilet, if relevant.)*  [IF NOT POSSIBLE TO DETERMINE, INDICATE THEY DO NOT KNOW. SHOW PICTURES, PROBE, AND READ ALOUD AS NEEDED.] | **Flush or Pour Flush Toilet to**  Piped Sewer System  Septic Tank  Pit Latrine  Somewhere Else  Don't Know Where  **Pit Latrine**  Ventilated Improved Pit Latrine  Pit Latrine with Slab  Pit Latrine with Sitting Toilet  Pit Latrine Without Slab/Open Pit  **Other**  Composting Toilet (EcoSan)  Container-based Toilet  Bucket Toilet  Hanging Toilet/Hanging Latrine  No Facility/Bush/Field  Other: _______________  Don’t know | 1  2  3  4  5  6  7  8  9  10  11  12  13  0  96  99 |  |
| D_02 | Where is this facility located? | Inside the compound  Less than 30m outside the compound  More than 30m outside the compound  Other: ____________  Don’t know | 1  2  3  96  99 |  |
| D_03 | How many squat holes (“doors”) does the toilet facility have?  *Write “99” if they do not know.* | _____________ squat holes |  |  |
| D_04 | Do you share this toilet facility with other households? | Yes  No  Don’t know | 1  0  99 | >>D_06  >>D_06 |
| D_05 | Including your own household, how many households use this toilet facility?  *Write “99” if they do not know.* | 3 households or less  Between 4 and 6 households  Between 7 and 10 households  More than 10 households  Don’t know | 1  2  3  4  99 |  |
| D_06 | Do you have to pay to use this toilet facility? | Yes  No  Don’t know | 1  0  99 | >>E_00  >>E_00 |
| D_07 | How often do you pay to use the facility? | Weekly  Monthly  Every time I use it  Other: _______________  Don’t know | 1  2  3  96  99 |  |
| D_08 | How much do you spend to use facility [FREQUENCY OF PAYMENT]? | Amount in KES:  [WRITE 99 IF THEY DO NOT KNOW THE AMOUNT.] | __ |  |

| **Q No.** | **Emptying services**  ***(And establish Factors D and E)*** | **Options/Answers** | |  | **Skip to** |
| --- | --- | --- | --- | --- | --- |
| E_00 | *CALCULATE NUMBER OF ONSITE SANITATION FACILITIES IN COMPOUND* | *If C_06 = 2-3-6-7-8-9-10 or*  *C_32 = 2-3-6-7-8-9-10* | | Number (N) | >>E_01 |
|  |  | Else | | 0 | >>E_13 |
| *The rest of this section only applies when there is an onsite toilet facility in the compound.*  *Repeat this section N times.* | | | | | |
| E_01 | When was the last time your toilet was emptied? | Never been emptied  In the last three months  Between 3 and 12 months ago  Between 1 and 2 years ago  Over 2 years ago  Don’t know | | 0  1  2  3  4  99 | >>E_13  >>E_12 |
| E_02 | What type of service provider conducted the emptying?  *(Show photos of small and large vacuum trucks)* | Large vacuum truck (7-10 m^3^)  Small vacuum truck (< 7 m^3^)  Canter truck with pump  Certified manual emptiers (e.g., Gasia Poa)  Illegal manual emptiers  Self  Other: ________  Don’t know | | 1  2  3  4  5  6  96  99 |  |
| E_03 | Who paid for these costs?  (*Probe to know whether each category contributed to the cost)* | Me / my family  Compound neighbors  Tenants  Landlord  Caretaker  Other: _________________  Don’t know | | Y/N  Y/N  Y/N  Y/N  Y/N  96  99 |  |
| E_03d | *IF ANSWERED “me” to E_03:*  In total, how much did the emptying cost? (including solid waste removal, if any)  *(Write 0 if none, 99 if don’t know)* | _________________ KES | |  |  |
| E_03e | *IF ANSWERED “me” AND “other tenants” to E_03:*  How many tenants shared these costs? | ______ # of tenants | |  |  |
| E_09 | *IF OWNER AND ANSWERED “tenants” to E_03:*  Did the tenants then deduct the amount of emptying services from rent? | Yes, entire amount  Yes, part of the amount  No  Don’t know | | 1  2  0  99 |  |
| E_10 | *IF TENANT/CARETAKER AND ANSWERED “me” to E_03:*  Did you then deduct the amount you paid for emptying from your rent? | Yes, entire amount  Yes, part of the amount  No  Don’t know | | 1  2  0  99 |  |
| E_11 | *FOR TENANTS:*  *CALCULATE TENANT CATEGORY (Factor D)* | *If C_54 = CAT 1* | | | |
|  |  | *If E_00 = NO* | | *Cat 1.0* | *>>F_01* |
|  |  | *Else If E_10 =1* | | *Cat 1.1* |  |
|  |  | *Else* | | *Cat 1.2* |  |
|  |  | *If C_54 = CAT 2* | | | |
|  |  | *If E_00 = NO* | | *Cat 2.0* | *>>F_01* |
|  |  | *If E_10 =1* | | *Cat 2.1* |  |
|  |  | *Else* | | *Cat 2.2* |  |
| E_12 | *FOR OWNERS:*  *CALCULATE OWNER CATEGORY (Factor D)* | *If C_55 = CAT 3* | | |  |
|  |  | *If E_00 = NO* | | *Cat 3.0* | *>>F_01* |
|  |  | *Else* | | *Cat 3.1* |  |
|  |  | *If C_55 = CAT 4* | | | |
|  |  | *If E_00 = NO* | | *Cat 4.0* | *>>F_01* |
|  |  | *Else* | | *Cat 4.1* |  |
| E_13 | *[If E_01 !=0 or 99]:*  On a scale of 1 – 5, how satisfied/unsatisfied were you with the emptying service? [USE GRAPHIC SCALE]  *Very satisfied means that the respondent has no complaints* | Very unsatisfied  Somewhat unsatisfied  Average  Somewhat satisfied  Very satisfied  Don’t know | | 1  2  3  4  5  99 |  |
| E_14 | *[If E_01 !=0 or 99]:*  What did you dislike about the emptying service? Anything else?  *(probe as needed; select all that apply)* | Too costly  Price changed after first negotiation  Pit not entirely emptied  Solid waste not removed  Spillages  Smell  Sludge buried onsite  Sludge disposed of in the open  Need to conceal from authorities  Other: __________  Don’t know | | 1  2  3  4  5  6  7  8  9  96  99 |  |
| E_15 | *Designate ‘responsible for sanitation emptying.’* | CAT 1.1  CAT 2.1  CAT 3.1  CAT 4.1 | | “RESP” |  |
|  |  | Else | | “NORE” |  |
| E_16 | *FOR OWNERS CAT 3.1 and 4.1 and TENANTS CAT 1.1 and 2.1 (“RESP”):*  *UNLESS E_02 = 1 or 2*  What prevented you from using a vacuum truck?  *Select all that apply.* | Hasn’t heard about this service  Doesn’t know how to contact  Accessibility issue – from the road to my house  Accessibility issue – to this neighborhood  Sludge is too thick  Too costly  They do not come quickly  They do not entirely empty the pit  Lack of cleanliness  Other: _________  Don’t know | | 1  2  3  4  5  6  7  8  9  96  99 |  |
| E_17 | *FOR OWNERS CAT 3.1 and 4.1 and TENANTS CAT 1.1 and 2. 1 (“RESP”):*  *UNLESS E_02 = 4*  What prevented you from using formal manual emptiers? (Gasia Poa, Vukasasa, Blue Stars)  *Select all that apply.* | Hasn’t heard about this service  Doesn’t know how to contact  Accessibility issue – from the road to my house  Accessibility issue – to this neighborhood  Sludge is too thick  Too costly  They do not come quickly  They do not entirely empty the pit  Lack of cleanliness  Other: _________  Don’t know | | 1  2  3  4  5  6  7  8  9  96  99 |  |
| E_18 | *FOR OWNERS CAT 3.1 and 4.1 and TENANTS CAT 1.1 and 2. 1 (“RESP”):*  Have you ever used another type of service provider to empty the pit? | Yes  No  Don’t know | |  | >>E_20  >>E_20 |
| E_19 | If so, which type?  *Select all that apply*  *(Show photos of small and large vacuum trucks)* | Large vacuum truck (7-10 m^3^)  Small vacuum truck (<7 m^3^)  Canter truck with pump  Certified manual emptiers (e.g., Gasia Poa)  Illegal manual emptiers  Self  Other: ________  Don’t know | | 1  2  3  4  5  6  96  99 |  |
| E_20 | On average, how often does your toilet get emptied? | Has been emptied only once or less  Once per year or more  Less than once per year  Don’t know | | 0  1  2  99 | >>E_23  >>E_21  >>E_22  >>E_23 |
| E_21 | Every how many months does your pit get emptied? *(99 if don’t know)* | ______________ months | |  |  |
| E_22 | Every how many years does your pit get emptied?  *(99 if don’t know)* | ________________ years | |  |  |
| E_23 | *FOR OWNERS CAT 3.1 and 4.1 and TENANTS CAT 1.1 and 2.1 (“RESP”):*  Do you think the toilet will need to get emptied in the next 3 months? | Yes  No  Don’t know | | 1  0  99 | >>E24 |
| E_23b | Do you intend to empty the toilet in the next 3 months? |  | |  |  |
| E_24 | Can you see fecal sludge in the pit? | Yes  No  Don’t know | | 1  0  99 |  |
| E_25 | *CALCULATE EMPTYING NEED* | *Based on answers to questions A_07, B_14, E_01, E_23, E_24* | | YES |  |
|  |  |  | | NO |  |
| E_26 | Is the slab stable enough for emptying by Gasia Poa?  **Currently in CommCare: IF C_20 = “1” or C_45 = “1, “GPABLE”, “NOGP”** | Yes  No  Cannot observe/don’t know | | 1  0  99 |  |
| E_27 | Is the toilet accessible to VTOs (within 50m of road)?  **Currently in CommCare: IF C_21 = “1” or C_46 = “1, “VTOABLE”, “NOVTO”** | Yes  No  Cannot observe/don’t know | | 1  0  99 |  |
| E_28 | *If (E_12=1.1 or 2.1) or (E_13=3.1 or 4.1) (“RESP”)*  *CALCULATE FACTOR E* | If E_25=YES | | | |
|  |  | If E_26=1 and E_27=1 | A-both | |  |
|  |  | If E_26=1 and E_27=0 | A-GP | |  |
|  |  | If E_26=0 and E_27=1 | A-VTO | |  |
|  |  | If E_26=0 and E_27=0 | B | |  |
|  |  | Else | B | |  |
| **REPEAT QUESTIONS E_01 to E_28 for second onsite toilet, if any** | | | | | |

| **Q No.** | **Final Categories** | **Options/Answers** |  | **Skip to** |
| --- | --- | --- | --- | --- |
| F_01 | *FOR TENANTS:*  *CALCULATE FINAL CATEGORY* | Concatenate [E_12, “-“, E_28] | |  |
| F_02 | *FOR OWNERS:*  *CALCULATE FINAL CATEGORY* | Concatenate [E_13, “-“, E_28] | |  |

| **Q No.** | **TENANTS (CAT 2)**  **WTP for access to sanitation option** | **Options/Answers** |  | **Skip to** |
| --- | --- | --- | --- | --- |
| H_01 | Now we would like to show you some different sanitation options.  READ DESCRIPTION AND SHOW PHOTOS OF SELECTED sanitation options.  ASK THE FOLLOWING FOR EACH OPTION PRESENTED. | | | |
|  | *Have you read the script and shown the graphics to the respondent?* | Yes  No | 1  0 |  |
| H_02 | Suppose this sanitation option was available in this compound.    Would you be willing to pay an additional X/month (randomized) on top of your rent? | Yes  No  Don’t know | 1  0  99 |  |
| H_03 | *[Y amount to be adjusted up or down based on H_02]*  Would you be willing to pay an additional Y/month on top of your rent? | Yes  No  Don’t know | 1  0  99 |  |
| H_04 | What is the highest additional amount you would be willing to pay per month on top of your rent for this sanitation facility to be present in this compound? | Amount:________________ |  |  |

| **Q No.** | **TENANTS (CAT 1.1, 1.2, 2.1, 2.2)**  **WTP for emptying service** | **Options/Answers** |  | **Skip to** |
| --- | --- | --- | --- | --- |
| I_01 | Now we would like to show you different types of emptying services.  READ DESCRIPTION AND SHOW PHOTOS OF SELECTED emptying services.  ASK THE FOLLOWING FOR EACH OPTION PRESENTED. | | | |
|  | *Have you read the script and shown the graphics to the respondent?* | Yes  No | 1  0 |  |
| I_02 | Suppose this emptying service was performed when necessary for the toilet facility in this compound.    Would you be willing to pay an additional X/month (randomized) on top of your rent? | Yes  No  Don’t know | 1  0  99 |  |
| I_03 | *[Y amount to be adjusted up or down based on I_02]*  Would you be willing to pay an additional Y/month on top of your rent? | Yes  No  Don’t know | 1  0  99 |  |
| I_04 | What is the highest additional amount you would be willing to pay per month on top of your rent for this emptying service to be performed when necessary for the toilet facility in this compound? | Amount:________________ |  |  |

| **Q No.** | **OWNERS (CAT 4-5)**  **WTP for toilet construction** | **Options/Answers** |  | **Skip to** |
| --- | --- | --- | --- | --- |
| J_01 | Now we would like to show you different sanitation options.  READ DESCRIPTION AND SHOW PHOTOS OF SELECTED SANITATION OPTIONS.  ASK THE FOLLOWING FOR EACH OPTION PRESENTED. | | | |
|  | *Have you read the script and shown the graphics to the respondent?* | Yes  No | 1  0 |  |
| J_02 | Suppose this sanitation facility was available for you to construct for a total amount of X (randomized) (including materials, transportation, and labor).  Would you be willing to pay X ? | Yes  No  Don’t know | 1  0  99 |  |
| J_03 | *[Y amount to be adjusted up or down based on J_02]*  Would you be willing to pay Y ? | Yes  No  Don’t know | 1  0  99 |  |
| J_04 | What is the maximum amount you would be willing to pay to construct this sanitation facility? | Amount:________________ |  |  |
| J_05 | Suppose there is an option to purchase this sanitation facility with 12 monthly payments of X (randomized) amount.  Would you be willing to pay X (randomized) monthly over 12 months? | Yes  No  Don’t know | 1  0  99 |  |
| J_06 | *[Y amount to be adjusted up or down based on J_05]*  Would you be willing to pay Y monthly over 12 months? | Yes  No  Don’t know | 1  0  99 |  |
| J_07 | What is the maximum you would be willing to pay to construct this sanitation facility (monthly over 12 months)? | Amount:________________ |  |  |
| J_08 | If you constructed this sanitation option, would you change the rent for your tenants? | Yes  No  Don’t know | 1  0  99 | >>  >> |
| J_09 | How much would you increase rent per month for your tenants? | Amount:________________ |  |  |

| **Q No.** | **OWNERS (CAT 1.1, 2.1, 3.1, 4.1)**  **WTP for emptying service** | **Options/Answers** |  | **Skip to** |
| --- | --- | --- | --- | --- |
| K_01 | Now we would like to show you some different emptying service options.  READ DESCRIPTION AND SHOW PHOTOS OF SELECTED EMPTYING SERVICES.  ASK THE FOLLOWING FOR EACH OPTION PRESENTED. | | | |
|  | *Have you read the script and shown the graphics to the respondent?* | Yes  No | 1  0 |  |
| K_02 | Suppose this emptying service was available to you for X (randomized) amount.  Would you be willing to pay X ?  *For sharing tenants, this would be equivalent to X/E_03e KES per tenant.* | Yes  No  Don’t know | 1  0  99 |  |
| K_03 | *[Y amount to be adjusted up or down based on K_02]*  Would you be willing to pay Y ?  *For sharing tenants, this would be equivalent to X/E_03e KES per tenant.* | Yes  No  Don’t know | 1  0  99 |  |
| K_04 | What is the maximum amount you would be willing to pay for this emptying service? | Amount:________________ |  |  |
| K_05 | Suppose there is an option for you to pay Gasia Poa/the Kisumu Waste and Waste Water Association (KWWWA) X (randomized)/month that makes you eligible to request 1 emptying service per year at any time you want it.  Would you be willing to pay X (randomized) per month for this service?  *For sharing tenants, this would be equivalent to X/E_03e KES per tenant per month.* | Yes  No  Don’t know | 1  0  99 |  |
| K_06 | *[Y amount to be adjusted up or down based on K_05]*  Would you be willing to pay Y per month?  *For sharing tenants, this would be equivalent to X/E_03e KES per tenant per month.* | Yes  No  Don’t know | 1  0  99 |  |
| K_07 | What is the maximum amount you would be willing to pay per month to subscribe to this emptying service? | Amount:________________ |  |  |
| K_08 | If you used this emptying service, would you add to your rent per month for your tenants? | Yes  No  Don’t know | 1  0  99 | >>  >> |
| K_09 | How much would you add to your rent per month for your tenants? | Amount:________________ |  |  |

| **Q No.** | **Water** | **Options/Answers** |  | **Skip to** |
| --- | --- | --- | --- | --- |
| P_01 | [*If Cat 2, 4, or 5*]  Which of the three sanitation options would you prefer? | Pour-flush to lined pit  Pour-flush to sewer  Container-based sanitation  None  Don't know | 1  2  3  0  99 |  |
| P_02 | Which emptying service do you prefer? | VTOs  Gasia Posa  Other manual (illegal) emptiers  Don't know | 1  2  3  99 |  |
| P_03 | How would you prefer to pay for a new toilet? | Lump sum payment (all at once)  In installments  Combination of some up front and the rest in installments  Other: _______________  Don't know | 1  2  3  96  99 |  |
| P_04 | How would you prefer to pay for emptying services? | Lump sum payment (all at once), at time of emptying  As a subscription  Combination of some in lump sum at time of emptying and some subscription payment  Other: _______________  Don't know | 1  2  3  96  99 |  |

| **Q No.** | **Water** | **Options/Answers** |  | **Skip to** |
| --- | --- | --- | --- | --- |
| L_01 | Is there a piped water connection on the premises of this compound? | Yes  No  Don’t know | 1  0  99 |  |
| L_02 | What is the main source of water for your household’s activities such as cooking and handwashing?  [SELECT ONE. PROBE AS NEEDED. IF HOUSEHOLD STATES WATER VENDOR, PROBE ON WATER SOURCE]. | **Piped Water**  Piped Into Dwelling  Piped To Yard/Plot  Piped To Neighbor  Public Tap/Kiosk  **Other**  Tube Well or Borehole  Dug Well  Protected Well  Unprotected Well  Protected Spring  Unprotected Spring  Rainwater harvesting  Tanker Truck  Cart With Small Tank  Surface Water (River/Dam/Lake/Pond/ Stream/Canal/Irrigation Channel)  Bottled Water  Other: _______________  Don’t know | 1  2  3  4  5  6  7  8  9  10  11  12  13  14  15  96  99 |  |
| L_03 | *ONLY ASK IF WATER SOURCE IS* ***NOT****:*   - *PIPED INTO DWELLING* - *PIPED TO YARD/PLOT*   *OTHERWISE, SKIP TO L_04*  Where is that water source located? | In Own Dwelling  In Own Yard/Plot  Elsewhere  Other: _______________  Don’t know | 1  2  3  96  99 | >>L_05  >>L_05 |
| L_04 | How long does it take to go there, get water, and come back? | Minutes: |  |  |
|  |  | Don’t know | 99 |  |
| L_07 | Do you have to pay to get water from this source? | Yes, a per-bucket fee  Yes, a flat monthly fee  Yes, a flat weekly fee  Yes, a utility bill  No  Other: _________  Don’t know | 1  2  3  4  0  96  99 | >>M_01  >>M_01  >>M_01  >>M_01  >>M_01  >>M_01 |
| L_08 | How much does a 20-L bucket of water cost? |  |  |  |

| **Q No.** | **Household Finances and Assets** | **Options/Answers** |  | **Skip to** |
| --- | --- | --- | --- | --- |
| M_01 | What is your household’s TOTAL income per month?  [PROBE FOR ALL INCOME SOURCES. READ RANGE.] | <3,000 KES  3,000 – <5,000 KES  5,000 – <7,000 KES  7,000 – <10,000 KES  10,000 – <23,000 KES  23,000 – <50,000 KES  50,000 – <120,000  ≥120,000 KES  Don’t know  Refuse to answer | 1  2  3  4  5  6  7  8  99  98 |  |
| M_03 | Does your household have …? | Electricity Y / N / DK  Radio Y / N / DK  Television Y / N / DK  Non-mobile phone/landline Y / N / DK  Refrigerator Y / N / DK  Solar panel Y / N / DK  Table Y / N / DK  Chair Y / N / DK  Sofa Y / N / DK  Bed Y / N / DK  Cupboard Y / N / DK  Wall clock Y / N / DK  Microwave oven Y / N / DK  DVD player Y / N / DK  Cassette or CD player Y / N / DK | |  |
| M_04 | Does any member of this household own a..? | Watch Y / N / DK  Mobile phone Y / N / DK  Bicycle Y / N / DK  Motorcycle/motor scooter Y / N / DK  Animal-drawn cart Y / N / DK  Car or truck Y / N / DK  Boat with a motor Y / N / DK | |  |
| M_05 | What type of fuel does your household primarily use for cooking? | Electricity  LPG/Natural gas  Biogas  Paraffin / Kerosene  Coal, lignite  Briquettes  Charcoal  Wood  Straw/shrubs/grass  Agricultural crop  Animal dung  No food cooked in household  Other: _______________  Don’t know | 1  2  3  4  5  6  7  8  9  10  11  0  96  99 |  |
| M_06 | How many rooms does this household occupy for living, eating, and sleeping?  *Do not count bathroom, kitchen, or toilet.* | Number of rooms: |  |  |
| M_07 | In what type of housing does your household live? [OBSERVE OR ASK] | House (single family), own compound, or bungalow  Multiple houses connected  Flat / Apartment  Servants Quarters  County houses, municipal houses  Shack, container, tent (Improvised home)  Single room in a compound house  Living quarters attached to shop/workplace  Other: _______________ | 1  2  3  4  5  6  7  8  96 |  |
| M_08 | Main material of the floor of respondent’s home | Earth/sand  Dung  Wood planks  Palm/Bamboo  Parquet or Polished Wood  Vinyl or Asphalt Strips  Ceramic Tiles  Cement  Carpet  Other: _______________  Don’t know | 1  2  3  4  5  6  7  8  9  96  99 |  |
| M_09 | Main material of the roof of respondent’s home | No Roof  Thatch/Grass/Makuti  Dung/Mud/Sod  Iron Sheets  Tin Cans  Asbestos Sheet  Concrete  Tiles  Other: _______________  Don’t know | 1  2  3  4  5  6  7  96  99 |  |
| M_10 | Main material of the walls of respondent’s home  *Covers more than 50% of the wall.* | No Walls  Cane/Palm/Trunks  Dung/Mud/Sod  Bamboo with Mud  Stone with mud  Uncovered adobe (mud bricks)  Plywood  Cardboard  Reused wood  Iron sheets  Cement  Stone with Lime/Cement  Bricks (kiln dried)  Cement Blocks  Covered Adobe (plastered mud bricks)  Wood planks/shingles  Other: _______________  Don’t know | 0  1  2  3  4  5  6  7  8  9  10  11  12  13  14  15  96  99 |  |
| M_11 | Does your household use M-Pesa? | Yes  No  Don’t know | 1  0  99 |  |

| **Q No.** | **CAT 1.1-A, 2.1-A, 3.1-A, 4.1-A**  **Emptying vouchers** | **Options/Answers** |  | **Skip to** |
| --- | --- | --- | --- | --- |
| N_01 | You have been randomly selected to receive a voucher for emptying services. We will be giving you this voucher, which is like a coupon, to allow you to get an emptying job at a cheaper price. <Demonstrate with voucher.> On this voucher, you can see what type of emptying service you are eligible to buy. Should you choose to get this service, you can see the price you’d be required to pay (no more, no less). You can also see the price that the emptying service normally costs. This voucher is only good until X date, 2019 and you are only able to redeem it with supplier______. This supplier is available every day from x-y___ and can be contacted through this number. Please remember that you are not required to buy the service. | | | |
|  | *Have you read the script and shown the voucher to the respondent?* | Yes  No | 1  0 |  |
| N_02 | *Which voucher discount rate did the respondent receive?* | _____________ |  |  |
| N_03 | *What is the voucher number on the voucher they received?* | __ __ __ ___ |  |  |
| N_04 | *Take a photo of the voucher* |  |  |  |

| **Q No.** | **CAT 4-5**  **Toilet vouchers** | **Options/Answers** |  | **Skip to** |
| --- | --- | --- | --- | --- |
| O_01 | You have been randomly selected to receive a voucher for constructing a toilet. We will be giving you this voucher, which is like a coupon, to allow you to buy a sanitation solution at a cheaper price. <Demonstrate with voucher.> On this voucher, you can see what type of sanitation solution you are eligible to buy. Should you choose to buy the solution, you can see the price you’d be required to pay (no more, no less). You can also see the price that the sanitation solution normally costs. This voucher is only good until X date, 2019 and you are only able to redeem it at ____storE______. This store is located ______ and is open ___everyday from x-y___. Please remember that you are not required to buy the sanitation option. | | | |
|  | *Have you read the script and shown the voucher to the respondent?* | Yes  No | 1  0 |  |
| O_02 | *Which voucher discount rate did the respondent receive?* | _____________ |  |  |
| O_03 | *What is the voucher number on the voucher they received?* | __ __ __ ___ |  |  |
| O_04 | *Take a photo of the voucher* |  |  |  |

| Text | **This concludes the survey. Is there anything else you’d like us to know?**  The interview is finished. Thank you very much! | Comments: |
| --- | --- | --- |
