# Supplementary material for: When pits fill up: Supply and demand for safe pit-emptying services in Kisumu, Kenya
Source: PLoS One. 2020 Sep 3;15(9):e0238003. doi: 10.1371/journal.pone.0238003 (PMC7470379; doi:10.1371/journal.pone.0238003)
Supplement: S2 Text — (DOCX) [file pone.0238003.s008.docx]

# **Household Survey: Pre-Screening**

# **Nonro mar udi**

**[INSTRUCTIONS TO THE INTERVIEWER: The following statement should be read to every potential interviewee.]**

**CHIK NE JAPENJ PENJO: wechegi ibiro som ne jaduok penjo**

May I have a minute of your time, please? My name is ______________. I am a staff member at [ORGANIZATION] based in [COUNTRY]. I would like to invite you as a head of household to participate in our research study. The purpose of our research is to understand the ability of households to pay for sanitation options in [CITY]and will be conducted over 5 months. You are being asked to participate in this study because you live in one of the areas selected for the present study.

**Bende inyalo miya dakika achiel mari, kiyie? Nyinga en_______________________. An jatich mar [ORGANIZATION] mayudore [COUNTRY]. Daher mar ruaki kaka wuon ot mondo igol pachi e nonro . Gimomiyo watimo nonro ni en ng’eyo nyalo mar ute mar chulo chenro mag ler e aluorawa mar [ CITY] ma ibirotim kuom dueche Abich(5). Okwayi ni mondo idonji e nonro nikech idak e aluora ma oyier e nonoro ni.**

Is this a good time to talk?

**Be mae en saa maber mar wuoyo kodi?**

Would you be willing to answer a few questions about yourself to find out if you might qualify for the study? You can stop at any time.

**Be diher duoko penjo matin ewiyi mondo okonywa ng’eyo ka inyalodonjo e nonro ni? *Oyieni wuok enonro samoro amora***

Thank you for your interest in our research study. May I begin?

**Erokamano kuom yie golo pachi ewi nonro wani. Be anyalo chako**?

**Pre-Interview Screening Questions –; REQUIRED TO BE OVER AGE 18, LIVING ,IN THE COMPOUND AND ONE OF THE HOUSEHOLD HEADS.**

1. Do you live on this compound?

**Be idakie odni?**

1. Are you over 18 years old?

**In gi igni apar gaboro kadhi nyime?**

1. Do you take part in the household’s financial decisions?

**Be igolo pachi kaluore gi weche pesa mitiyo go e ot?**

**If subject qualifies (answers “Yes” to pre-screening), then continue to CONSENT below.** If not, thank them again for their time and interest.

IF RESPONDENT CONSENTS BUT IS UNABLE TO SPEND TIME, ASK: Is there a more convenient time when I could ask you some questions? **RECORD IN COMMCARE.**

**KA JADUOK PENJO ODICH, to penji ka ntie seche moko ma obroyudo thuolo ma apenje penjo gi?**

Household Survey: Consent for Interview

**Timo nonro mar ot: yie mar donjo e nonro ni**

The interview will involve questions about living conditions in [CITY], access to and satisfaction with sanitation services. The interview should last no longer than 1 hour or until you feel that you have told me everything you want me to know. If you agree to participate in this research, I will conduct an interview with you now.

**Tuakgi biro bedo e wi yore mag dak e [ city] , yudruok to gi bedo mamor e weche ler . Tuakgi biro bedo mok ng’eny ne saa achiel kata nyaka i winj ni esekona gigo duto ma iduaro ni ang’e. ka iyie donjo e nonro ni, a biro tuak kodi sani.**

There are no right or wrong answers, so please be honest and tell us what is true for you. Information from this study may help increase understanding and awareness of what it is like to live in [CITY]. There are no personal risks or benefits to your participation. Everything that you say will be confidential, and we will not use your real name or any identifying information in any of our reports or papers. Some staff of the Aquaya Institute may sometimes look at your record for research purposes. The results will be used to inform government actors and companies about the ability to pay for existing sanitation costs.

**Onge duoko man kare kata mok kare, koro kiyie to tem ichiwo a diera ma kor ka kori. Paro mawuok e nonro ni nyalo konyo medo winjo gi ng’eyo kaka chal mar dak e[city] en. Ong’e inyruok moro a moro kata chudo mibiro yudo kowuok kuom donjo e nonro ni. Gimoro a mora miwacho ibiro kan kiling ling ,kendo nyingi madier okbi tigo kata gimoro amora motenore kodi e golo duoko kata keto e oboke. Jo tij Aquaya Institute moko samoro nyalo ng’iyo duoko magi kagiduaro tiyo go e yor nonro. Duoko mar nonro ni ibirotigo kuom konyo sirikal koda kembe moko kod paro eyo ma inyalochulgo chenro mag ler mar aluora madhi nyime sani.**

Your participation in this research is completely voluntary. You can decline to answer any questions, and if you do not wish to continue, you can withdraw from the study at any time for any reason. You will not receive any monetary payment for this questionnaire. An alternative is to not participate in this study.

s

**Donjo ni e nonro ni en chiwruok mari. Inyalo tamori duoko penjo moro a mora to kokiduar dhi nyime, inyalo wuok e nonro ni samora a mora. Okibiyudo yor chudo moramora motenore gi penjogi. E yo machielo en ni inyalo weyo donjo e nonro ni.**

If you have any questions or concerns about the research, please feel free to contact me. I can be reached at [phone number] or [email address].

**Ka in kod penjo kaluwore kod nonro ni, in gi thuolo mar tudori koda. Anyalo yudora:[Namba Simu] kata[Email]**

## If you agree to voluntarily participate in this study, please say so.

**Ka iyie timo nonro ni to wachi**

[ALL QUESTIONNAIRES WILL BE SAVED BY THE INTERVIEWER REGARDLESS OF THE RESPONDENT’S DECISION TO PARTICIPATE OR NOT TO PARTICIPATE.]

PENJO TE IBROKAN GI JAPENJO KATA KA JADUOK {PENJO ODUOKO KATA KA OK ODUOKO

_____________________ _____________________ ___________________

Participant Name Participant Signature Date

**Nying jalno magolo pache**  **Sei mar jalno magolo pache Tarik**

_____________________ _____________________ ___________________

_____________________ _____________________ ___________________

Interviewer Name Interviewer Signature Date

**Nying jalno matimo nonro Sei mar jalno matimo nonro Tarik**

This research has been reviewed and approved by the [AMREF]Institutional Review Board. If you have any questions about your rights as a research participant, you can contact their office: [PHONE], [EMAIL], or [ADDRESS].

**Chenro ni osepuodhi gi migawo matimo puoth marwa. Ka in kod penjo kaluwore kod ratiro mari kaka jalno magolo pache e nonro ni, inyalotudori gi ofis:[Namba Simu],[Email]**

Household Survey: CommCare

**Penjo mag ute: CommCare**

| **Question**  **Penjo** | **Answer**  **Duoko** | **Question**  **Penjo** | **Answer**  **Duoko** |
| --- | --- | --- | --- |
| Date:  **Tarik** |  | Time Interview Began **Saa mochakore** |  |
| A_01 Interviewer Name N**ying ja penjo** | Enumerator 1­­­­­­­­­­­­­­__1  Enumerator 2 ___2  Enumerator 3___3  Enumerator 4_____4  Enumerator 5_____5  Supervisor ____6  Others______7 | Time Interview Ended  **Saa morume** |  |
| A_01b  What number is this compound of those ypu have visited today  **Ma ot namba adi miselimo kawuono?** | | 1^st^ compound of the day  **ot mokuongo e odiechieng**  2^nd^ compound of the day  **ot marariyo e odiechieng**  3^rd^ compound of the day  **ot maradek eodiechieng**  4^th^ compound of the day  **Ot maraguen e odiechieng**  5^th^ compound of the day  **Ot marabich eodiechieng**  6^th^ compound of the day  **Ot marauchiel eodiechieng**  7^th^ compound of the day  **Ot marabiriyo e odiechieng**  8^th^ compound of the day  **Ot maraboro eodiechieng**  9^th^ compound of the day  **Ot marochiko e odiechieng** | 1  2  3  4  5  6  7  8  9 |

| **Q No.**  **Namba mar penjo** | **Pre-interview screening**  **Penjo mabiro mokuong’o** | **Options/Answers**  **Duoko** | |  | | **Skip to kadhi nyaka** |
| --- | --- | --- | --- | --- | --- | --- |
| A_01 | Take GPS measurement | Lat: ________________. Long:________________. | | A_11 | | Take GPS measurement |
| A_02 | Is a household head available to be interviewed who lives here and is ≥18 years old?  **Be wuon ot ma odak kae ntie ma en ja higni 18 kadhi nyime?** | Yes **kamano**  No **oyo** | | 1  0 | | >>A_03 |
| A_03 | READ CONSENT FORM SOM OBOKE MAR YIE DONJO ENONRO  Are you willing to participate in the study?  **Be iyie donjo e nonro ni?** | Yes **kamano**  Yes, though at a later date **kamano, lakini chieng machielo**  No **oyo** | | 1  2  0 | | >>A_05  >>End **Giko** |
| A_04 | How many times have you visited this Compound?  **Iselimo kar dak ni didi?** | _______ | |  | | >>End if **Giko ka**>2 |
| A_05 | May a household head who lives here and is ≥18 years old be available at a later time?  **Be ntiero wuon ot ma odak kae ma nyalo yudore bang’e?** | Yes **kamano**  No **oyo** | | 1  0 | | >>End **Giko** |
| A_06 | When would be a good time to return?  **En saa mane maber ma onyalo duoge?** | __________________ | |  | | >>Return  **DUOKI** |
| A_07 | Name of LIA  **Nying LIA** | Bandani  Manyatta A  Manyatta B  Nyabera  Nyalenda A  Nyalenda B  Nyamasaria  Obunga  Otonglo | | 1  2  3  4  5  6  7  8  9 | |  |
| A_08 | Name of respondent  **Nying jalno maduoko penjo** | |  | | | |
| A_09 | Phone number of respondent  **Namba mar sime mar jaduok penjo** | |  | | | |
| A_10 | Do you know if this respondent is a tenant or a landlord?  **Be ing’eyo ka jaduok penjo en landlord kata japang’o?** | | Yes, landlord/homeowner  Yes, tenant  Don’t know | | | |
| A_09 |  | | L | | | |
| A_12 | Compound number  **Namba mar kompaund** | | Enumerator # month-day  **Japenj penjo# due-tarik**  Number .Owner status  **Namba .wuongi chalne** | |  |  |
|  |  | |  | | 1  2  96 |  |
| IneligibleB  **mokoyier** | If the household was not eligible, why were they ineligible?  **Ka ne odni okoyier ne nenro, ang’o momiyo** | | No one was home after 3 tries.  **Ne ong’e ng’ato e dala bang kaselimo didek.**  Household was not willing to participate.  **Jo ot neokdwar donjo e nonro**  Household head was not over 18 years old.  **Wuon ot ne okoromo igni 18**  Other: _**mamoko**_______ | | 1  2  3  96 |  |

| **Q No.** | **Demographics** | **Options/Answers** |  | **Skip to** |
| --- | --- | --- | --- | --- |
| B_01 | Does the respondent know their birthdate?  **Be jaduok penjo ong’eyo ndalo mane onyuole** | Yes **kamano**  No **oyo** | | >>B_03 |
| B_02 | What is the birthdate of the respondent? **Jaduog penjo nonyuol karang’o?**  *If they don’t know their birth month, keep the day of the survey (i.e., February 12) and change the year.* ***Ka poni gikia dwe mane onyuolgi, ket tarik man sama itimo e nonro ni(kaka februari 12) kasto lok iga*** | __ __ / __ __ / __ __ __ __  DAY **odiechieng** / MONTH **dwe**/ YEAR **iga** | |  |
| B_03 | What is the approximate age of the respondent?  **Iga mar jaduok penjo nyalo bedo adi?** | Years: igni: | |  |
| B_04 | What is the gender of the respondent?  **Jaduok penjo en chuech mane?** | Male **dichuo**  Female **miyo** | 1  2 |  |
| B_05 | Education level completed by respondent  **Jaduok penjo osomo marom nade?** | None **onge**  Pre-primary **Nasari**  Primary **praimari**  Post-primary training (e.g. vocational, tailoring, masonry) **Tiegruok mag tije luedo**  Secondary **Sekondari**  Mid-level collegekar **Tiegruok man diere**  University **Mbalariany**  Post-graduate  **Tat sosmo man malo**  Other: **mamoko**_______________  Refuse to answer **otamore duoko**  Don’t know **okia** | 0  1  2  3  4  5  6  7  96  98  99 |  |
| B_06 | What is your marital status?  **Chalni mar kend chalnade?** | Married/In Union **osekendi/isekendo**  Widowed  **Chi liel/ chuor liel**  Separated/Divorced **Upogoru**  Single **Pok okendi/ikendo**  Other: **mamoko** _______________  Refuse to answer **otamore duoko** | 1  2  3  5  96  99 |  |
| B_07 | How many people are in your household? (Number ofPeople who eat and sleep here more than 50% of the time in the last 12 months)  **Gin ji adi mantiere e odi? ( joma chiemo kendo nindo ka moingo atam 50% mar seche gi e dweche apar gariyo mokalo)** | Adults (18+ years, including respondent) **Joma dongo ma ikgi 18+ kimendo nyaka jaduok penjo** |  |  |
| B_08 |  | Children 5-<18 years  **Nyithindo ma igni 5< 18** |  |  |
| B_09 |  | Children 0-4 years  **Nyithindo ikgi 0-4** |  |  |
| B_10 | Does anyone in your household have difficulty walking or moving around? (e.g., elderly, persons with disability) **Be ng’ato a ng’ata ma wuok e odni nigi ng’ol mora mora?( ng’a t ma oti, ng’a mangi ngol)** | Yes **kamano**  No **oyo**  Refuse to answer **otamore duoko** | 1  2  99 |  |
| B_11 | How many years have you lived in this compound?  **Isedak e odni kuom igni adi?** | Lived here <1 year  **Matin ne iga achiel**  Lived here ≥1 year: LIST YEARS **Mageny ne iga achiel: ndik igni-**__  Don’t know **okia** | 1  2  99 |  |
| B_12 | Do you own your dwelling?  **In wuon kama idake ni?**  *[If caretaker, asks if he pays rent or not](* ***ka en jarit landi, penje ka be ochulo rent koso okochul)*** | Yes, own **kamano, en mara**  No, rent **oyo, a kodesa**  Housed for free **Adak nono**  Other: **mamoko**_______________ | 1  2  3  96 |  |
| B_13 | Does anyone live in this compound outside of your household?  **Be nitiere nga’t mora mora modak kar kad/ dala ka ma ok jaodni?** | Yes **kamano**  No **oyo** | 1  0 | >> B_18 |
| B_14 | Other than your household, how many households live in this compound (including the landlord if any)?  **Kopogore gi odi, gin ute adi mantiere e plodni?( med wuon landi kantie)?** | Households udi |  |  |
|  |  | People**Jii**  *[ONLY ASK IF B_12<4]* |  |  |
| B_16 | *[If B_12 = 1]*  Do you own any of these other houses?  **Be in wuon ute mamoko gi?** | Yes **kamano**  No **oyo** | 1  0 | >> C_01 |
| B_17 | How many of these other houses do you own?  **Gin ute adi mamagi?** | Households **ute** | __ |  |
| B_18 | On average, what is the duration of rent for your tenants?  **Kiparo jodak gi dak ka marom nade?** | Less than 1 year  1-3 years  More than 3 years  Don’t know | 1  2  3  99 |  |
|  |  |  |  |  |
|  |  |  |  |  |
| B_16 |  |  |  |  |
| B_19 | How much rent do you collect every month from all your tenants in this compound ?  **En pesa adi michoko kuom jomodak e uteni dwe ka dwe?** | Don’t know **okia** | 1  2  3  4  99 |  |
| B_20 | *[If B_12=0 or 2]:*  Who owns this compound?  **Wuon ute gi/ dala en nga’?** | Private landlord  **weg ute mochuk kendi**  Government, county, railways **sirikal mamalo, sirikal mar kaunti**  Church or community organization  **kanisa kata riwruok mar jogueng**  Other: **mamoko**__________  Don’t know **okia** | 1  2  3  96  99 |  |
| B_21 | *[If B_12=2]:*  How much rent does your household pay every month?  *(99 for don’t know or refuse to answer)*  **Odni uchulo pesa adi dwe ka dwe?( 99 ka okia kata otamore duoko)** | Amount KES---------- **PESA R**  Refused to answer **otamore duoko**  Don’t know **okia** | 99 |  |

| **Q No.** | **Sanitation Facilities**  ***(Establish Factors B and C)*** | **Options/Answers** | | |  | **Skip to** |  |  |
| --- | --- | --- | --- | --- | --- | --- | --- | --- |
| C_01 | *FOR TENANTS: MAR JODAK*  Do you have access to a toilet in this compound ? **Kama idak e ni be une gi choo?** | Yes **kamano**  No **oyo** | | | 1  0 | >>_18 |  |  |
| C_02 | *FOR LANDLORDS/HOMEOWNERS:*  ***NE WEG UDI/ DALA***  Do you own a toilet in this compound?  **Be ingi choo e ute ni gi/ dala la ni?** | Yes **kamano**  No **oyo** | | | 1  0 | >>C 25 |  |  |
| C_03 | Do you share this toilet facility with other households?  **Be uriwo choo ni gi ute moko?** | Yes **kamano**  No **oyo** | | | 1  0 | >>C_05 |  |  |
| C_04 | Including your own household, how many households use this toilet facility?  **Koriwore nyaka odi, gin ute adi matiyo gi choo ni?** | Number of households  **Namba mar ut** | | | __ |  |  |  |
| C_05 | May I see this toilet?  **Be anyalo neon choo no?** | Yes **kamano**  No **oyo** | | | 1  0 |  |  |  |
| C_06 | What kind of toilet facility is it?  **En choo machal nade?**  [IF PERMISSION TO OBSERVE WAS NOT GRANTED, ASK RESPONDENT TO DESCRIBE, SHOW PHOTOS].  **KA OKOYIENI NENO, NYIS JADUOK PENJO MONDO OPIMNI, TAGNE PICHNI** | **Flush or Pour Flush Toilet to Ma iolo e pi**  Piped Sewer System **Ma otudi e siwage**  Septic Tank **Ma nigi septic**  Pit Latrine **Ma okuny matut to iguome**  Somewhere Else kamachielo  Don't Know Where okia  **Pit Latrine**  Ventilated Improved Pit Latrine **Choo ma okuny matut to ntie kama yamo donjo go**    Pit Latrine with Slab **Choo ma okuny to olosne dierot mar simiti**    Pit Latrine with Sitting Toilet **Choo ma okuny matut to ntie kama ji bede**  Pit Latrine Without Slab/Open Pit **Choo ma okuny matut to onge die rot mar simiti**  **Other mamoko**  Composting Toilet (EcoSan) **ekosan**  Container-based Toilet  **Choo mar kontaina**    Bucket Toilet **choo mar ndoo**  Hanging Toilet/Hanging Latrine **choo molier**  No Facility/Bush/Field **Ji konyore e bungu**  Other: **mamoko** _______________ | | | 1  2  3  4  5  6  7  8  9  10  11  12  13  0  96 |  |  |  |
| C_08 | How many squat holes (“doors”) does the toilet facility have?  **Gin dhoudi adi miguome ma choo ni nigodo?** | _____________ squat holes  **Bugni miguome** | | |  |  |  |  |
| *QUESTIONS C_06 THROUGH C_14 ARE OBSERVATIONS* ***PENJO C_06 NYAKA C_14 GIN MINENO*** | | | | | | |  |  |
| C_09 | Did you observe this toilet during a previous survey?  **Be ning’iyo choo ni e nonro mokalo?** | Yes **kamano**  No  **oyo** | | | 1  0 | >>C_23 |  |  |
| C_10 | What is the material of the toilet/ latrine walls?  *Cover more than 50% of the wall*  **En matiriol mane mar korot choo ni?**  ***Ma omako atama 50 mar korot*** | | Reed/grass **lum**  Mud/earthen **chuodho**  Mix of cement and mud simiti **moriw gi loo**  Brick **matafare**  Wood **yien**  Sheet metal **mabati**  Cement block **blok mar simiti**  Plastic **plastik**  No walls **onge korot**  Other: **mamoko** ________________  Cannot observe/don’t know **okanyal neno/ okia** | | 1  2  3  4  5  6  7  8  0  96  99 |  |  |  |
| C_11 | What is the material of the toilet/ latrine roof?  **En matiriol mane mar witado mar choo ni?** | | Reed/grass **lum**  Brick/clay tile **Taels**  Wood **yien**  Sheet metal **mabati**  Plastic **plastik**  No roof **onge tado**  Other:**mamoko** ________________  Cannot observe/don’t know **okanyal neno/ okia** | | 1  2  3  4  5  0  96  99 |  |  |  |
| C_12 | What is the material of the toilet/ latrine floor?  **En matiriol mane mar dierot choo ni?** | | Mud/earthen **chuodho**  Brick **matafari**  Wood **yien**  Cement **simiti**  Ceramic pan + concrete **bakul moket gi simiti**  No flooring / open pit onge **dierot/ choo ma pap**  Other: **mamoko**________________  Cannot observe/don’t know **okanyal neno/ okia** | | 1  2  3  4  5  0  99 |  |  |  |
| C_13 | What is the condition of the toilet / latrine floor?  **Dierot mar choo ni chalnade?** | | Spotless **ler paa**  Pretty clean, minimal dirt  **Ler, nigi buru matin**  Visible dirt or other trash  **Buru manenore kata lil mamoko**  Filthy – covered in dirt/trash/feces  **Ochido – nigi buru/ chilo/ minyaga**  Unbearably dirty – I would not use  **ochido kabisa – okanyal tiyogo**  Cannot observe/don’t know okanyal neno/ okia | | 1  2  3  4  5  99 |  |  |  |
| C_14 | Is the toilet/ latrine door lockable from the inside?  **Dhoot mar choo ni be lorore gi iye?** | | Yes kamano  No oyo  No door ong’e dhoot  Cannot observe/don’t know okanyal neno/ okia | | 1  2  3  99 |  |  |  |
| C_14a | What type of lock does the door have? **Dhood ni nigi lock machalnade?** | | String  Metal wire  **waya mar chuma**  Metal slide lock  **chuma midhiro to lorore**  Key lock  **miloro gi key**  Other: **mamoko** ________________ | | 1  2  3  4  96 |  |  |  |
| C_15 | Is there a waste receptacle in or near the toilet/latrine?  **Be nitiere kama iketo yugi e choo kata machiegni gi choo?** | | Yes **kamano**  No **oyo**  Cannot observe/don’t know **okanyal neno/ okia** | | 1  0  99 |  |  |  |
| C_16 | Is there a handwashing facility in the vicinity of the toilet (less than 10 meters)?  **Be nitiere gima iluoke luedo machiegni gi choo? ( matine mita 10)** | | Yes **kamano**  No **oyo**  Cannot observe/don’t know **okanyal neno/ okia** | | 1  0  99 |  |  |  |
| C_17a | Is there water at the handwashing facility?  **Benitiere pii kuma iluoke luedo?** | | Yes **kamano**  No **oyo**  Cannot observe/don’t know **okonyal neno/oki**a | | 1  0  99 |  |  |  |
| C_17b | Is there soap at the handwashing facility?  **Benitiere sabun kama iluoke luedo?** | | Ye  **kamano**  No **oyo**  Cannot observe/don’t know **okonyal neno/ okia** | | 1  0  99 |  |  |  |
| C_18 | Does the toilet have the following features?  **Be choo ni nigi gik machal kama?** | | Seat **kom**  Handrail **kar luedo**  Steps **steps**  **Lighting** ler | | Y/N  Y/N  Y/N |  |  |  |
| C_19 | IF TOILET NOT CONNECTED TO SEWER  Is there a manhole for desludging?  *(Select NO if Emptying requires breaking the slab) bentiere otuchi michodho go choo?* ***( yier Oyo ka chodho choo nyaka imuk dierot*** | | Yes **kamano**  No **oyo**  Cannot observe/don’t know**okanyal neno/okia** | | 1  0  99 |  |  |  |
| C 20 | IF TOILET NOT CONNECTED TO SEWER :  Is the slab stable enough for emptying by Gasia Poa?  KA CHOO OKOTUDI E SIWEJ  **Be chooni nigi dierot motegno minyalo chodhi gi Gasia Poa**  *Jo penj penjo ibiro yudo puonj ka* | | Yes **kamano**  No  **Oyoo**  Cannot observe /don’t know **okonyal neno/okia** | |  |  |  |  |
| C 21 | IF TOILET NOT CONNECTED TO SEWER :  Is the toilet accessible to VTO’s ( witjhin 50M of road?  **KA CHOO OKOTUDI E SIWEJ**  **Be chooni nyalo chop ma chodhi gi VTO ( entiere ma okbor gi mita 50 kiwuok e ndara**? | | Yes **kamano**  No  **Oyoo**  Cannot observe /don’t know **okonyal neno/okia** | |  |  |  |  |
| *END OF OBSERVATION* ***GIKO MAR NENO*** | | | | | | |  |  |
|  |  |  | | |  |  |  |  |
|  |  |  | | |  |  |  |  |
|  |  |  | | | __ |  |  |  |
| C_22 | *CALCULATE SANITATION QUALITY*  **PIM OKANG MAR LER MAR CHOO** | *If toilet is in the compound, improved, and with 3max households per squat hole* | | | HIGH |  |  |  |
|  |  | *Else or no toilet* | | | LOW |  |  |  |
| C_23 | Who paid for the toilet to be constructed? (CAN CIRCLE MULTIPLE) **ng’a mane mano chulo pesa mar gero choo?( OYIENI YIERO MATHOTH)** | Me / my family an/ **jo oda**  Landlord wuon plot  Compound neighbors (how many): **jirande ga (adi)**_____  Other: **mamoko** _________________  Don’t know **okia** | | | 12  3  96  99 |  |  |  |
| C_24 | *If* C_23 *=1:*  Where did you get money to construct the toilet? Ni yudo pesa mane I gero go choo kowuok kanye? | My own savings  **pesa na ma akano**  Borrowed from family/friends **pesa ma aolo kuom anyuola kata osiepe**  On credit **gowi**  Other: **mamoko** __________________ | | | 1  2  3  4 |  |  |  |
| C_25 | *If* C_23 *=1:*  , How long did it take you/your household to finish the payments?  **,** Nokawi in/ joodi **kinde marom nade mondo itiek chudo?** | No time - paid immediately  **onge saa- nachulo sechego**  Under one month  **matine dwe achiel**  1-6 months kind  **dwe achiel nyaka auchiel**  Over 6 months  **moingo dweche auchiel**  Other: **mamoko**__________________ | | | 1  2  3  4  5 |  |  |  |
| C_26 | Are there any members of this household that usually don’t use this toilet facility?  **Bende nitiere joodi moro a mora ma ok tigi choo ni?** |  | | | 1  0 |  |  |  |
| C_27 | On a scale of 1 – 5, how satisfied are you with this toilet? [USE GRAPHIC SCALE] e wi ratil 1-5,  **iyie marom nade gi choo ni? ( tigi ratil mar graf)** | Very unsatisfied **ok ayiego ahinya**  Somewhat unsatisfied **ok ayiego**  Average **ediere**  Somewhat satisfied **ayiego matin**  Very satisfied **ayiego ahinya**  Don’t know **okia** | | | 1  2  3  4  5  99 | >>C_25 |  |  |
| C_2 | What do you dislike about this toilet? Anything else?  **Ang’o ma okihero kuom choo ni?, gimachielo?**  *(probe as needed; select all that apply)* | Don’t like the type of toilet **okahero aina mar choo ni?**  Dirty **olil**  Bad smell **odung marach**  Health risk  **okober gi gima**  Uncomfortable  **oonge gi komfot**  Not suitable for children or elderly  **ok ober gi nyithindo kata joma osetii**  risk of falling **inyalo luar**  No privacy  **ong’e pandruok**  Inconveniently located  **en kama okber koda**  Security concerns **kwe ma kanyo**  No water **onge pii**  No receptacle for sanitary pads **onge kar keto gige mine (pads)**  Conflict with neighbors  **koko gi jirande**  Pit is full **choo opong**  Pit floods/ overflows during rainy season  **choo pong / pukore oko ndalo koth**  Blockages **odinore**  Other: **mamoko**_______  Don’t know **okia** | | | Y/N  Y/N  Y/N  Y/N  Y/N  Y/N  Y/N  Y/N  Y/N  Y/N  Y/N  Y/N  Y/N  96  99 |  |  |  |
| *FOR LANDLORDS/HOMEOWNERS: if doesn’t own toilet or the first toilet doesn’t serve all households he is responsible for*  *[If C_16< Number of tenants on compound+1 OR C_22=0]:*  *MAR WEG LOWO/UDI: ka ong’e gi choo katachoo mokuongo ok tigo gi ute te ma mage{ ka C_16 < namba mar jopango e landi + 1KATA C_22=0}* | | | | | | |  |  |
| C_29 | Do you own another toilet facility in this compound?  **Be in gi choo machielo e plot ka?** | Yes **kamano**  No **oyo** | | | 1  0 | >>C_40 |  |  |
| C_30 | How many households use this toilet facility (including your household if applicable)?  **Gin ute adi matiyo gi choo ni ( med nyaka mari kapo ni utiyo kode)?** | Households **ute** | | | ___ |  |  |  |
| C_31 | May I see this toilet?  **Be anyalo neno choo no?** | Yes **kamano**  No **oyo** | | | 1  0 |  |  |  |
| C_32 | What kind of toilet facility is it?  **En choo machalo nade?**  [IF PERMISSION TO OBSERVE WAS NOT GRANTED, ASK RESPONDENT TO DESCRIBE, SHOW PHOTOS].  **KA OKOYIENI NENO, NYIS JADUOK PENJO MONDO OPIMNI, TAGNE PICHNI** | **Flush or Pour Flush Toilet to Choo ma iolo e pii**  Piped Sewer System  **Ma otudi e pipe**  Septic Tank **Tangi ma okuny e loo**    Pit Latrine **Choo ma okunya matut**  Somewhere Else **Kama chielo**  Don't Know Where okia ni en kanye  **Pit Latrine** choo mokuny matut  Ventilated Improved Pit Latrine **Choo mokuny matut man gi kar muya**  Pit Latrine with Slab  **Choo ma okuny matut ma dier ode olos gi simiti**  Pit Latrine with Sitting Toilet **Choo ma okuny to nigi kar bet**  Pit Latrine Without Slab/Open Pit **Choo ma okuny matut to onge gi die rot mar simiti**  **Other mamoko**  Composting Toilet (EcoSan) **ekosan**  Container-based Toilet  **choo mar kontaina**  Bucket Toilet **choo mag ndoo**  Hanging Toilet/Hanging Latrine **choo ma olier**  No Facility/Bush/Field  **Jii konyore e bungu**  Other: mamoko _______________ | | | 1  2  3  4  5  6  7  8  9  10  11  12  13  0  96 |  |  |  |
| C_33 | How many squat holes does (“doors”) the toilet facility have?  **Gin dhoudi adi miguome ma choo ni nigodo?** | _____________ squat holes **bugni miguome**---- | | |  |  |  |  |
| *QUESTIONS C_35 THROUGH C_46 ARE OBSERVATIONS* ***PENJO C_35 NYAKA C_46 GIN MINENO*** | | | | | | |  |  |
| C_34 | Did you observe this toilet during a previous survey?  **Be ning’iyo choo ni e nonroni mokalo?** | Yes **kamano**  No **oyo** | | | 1  0 | >>C_54 |  |  |
| C_35 | What is the material of the toilet / latrine walls? *Cover more than 50% of the wall*  **En matiriol mane mar korot choo ni ?**  ***Maomako korot madirom ata 50*** | Reed/grass **lum**  Mud/earthen **chuodho**  Mix of cement and mud  **simiti moriw gi loo**  Brick **matafari**  Wood **yien**  Sheet metal **mabati**  Cement block **blok mar simiti**  Plastic **plastik**  No walls **onge korot**  Other: **mamoko** ________________  Cannot observe/don’t know **okanyal neno/ okia** | | | 1  2  3  3  4  5  6  7  0  96  99 |  |  |  |
| C_36 | What is the material of the toilet/ latrine roof? En **matiriol mane mar witado mar choo ni ?** | Reed/grass **lum**  Brick/clay tile **matafari/ tael**  Wood **yien**  Sheet metal **mabati**  Plastic **plastik**  No roof **ong’e tado**  Other: _**mamoko** _______________  Cannot observe/don’t know **okanyal neno/ okia** | | | 1  2  3  4  5  0  96  99 |  |  |  |
| C_37 | What is the material of the toilet /latrine floor?  **En materiol mane mar dierot choo ni ?** | Mud/earthen **chuodho**  Brick **matafare**  Wood **yien**  Cement **simiti**  No flooring / open pit  **ong’e dierot/ choo moyaw**  Other: mamoko________________  Cannot observe/don’t know **okanyal neno/ okia** | | | 1  2  3  4  5  6  99 |  |  |  |
| C_38 | What is the condition of the toilet / latrine floor?  **Dierot choo ni chal nade?** | Spotless **ler paa**  Pretty clean, minimal dirt  **ler, nigi buru matin**  Visible dirt or other trash  **buru manenore kata lil mamoko**  Filthy – covered in dirt/trash/feces  **ochido-nigi buru/chilo/minyaga**  Unbearably dirty – I would not use ochido kabisa- **okanyal tiyogo**  Cannot observe/don’t know **okanyal neno/okia** | | | 1  2  3  4  5  99 |  |  |  |
| C_39 | Is the toilet/latrine door lockable ?  **Dhoot choo ni be lorore ?** | Yes from the inside only **kamano gi iye kende**  Yes, from the outside ony  **kamano, gi okone kende**  Yes from both the inside and outside  **kamano, gi iye gi oko**  No **oyo**  No door **ong’e dhoot**  Cannot observe/don’t know **okanyal neno/ okia** | | | 1  2  3  99 |  |  |  |
| C_40 | Is there a waste receptacle in or near the toilet? **Be nitiere kama iketo yugi e choo kata machiegni gi choo?** | Yes **kamano**  No **oyo**  Cannot observe/don’t know **okanyal neno/ okia** | | | 1  0  99 |  |  |  |
| C_41 | Is there a handwashing facility in the vicinity of the toilet (less than 10 meters)?  **Be nitiere gima iluoke luedo machiegni gi choo ( matine mita 10)?** | Yes **kamano**  No **oyo**  Cannot observe/don’t know  **okanyal neno/okia** | | | 1  0  99 | >>c 43  >>c 43 |  |  |
| C 42a | Is there water at the handwashing facility?  **Benitiere pi e gir luoko luedo?** | Yes  **kamano**  No  **oyo**  Cannot observe/ don’t know  **okonyal neno /okia** | | |  |  |  |  |
| C_42b | Is there soap at the handwashing facility?  **Benitiere sabun e gir luoko luedo** | Yes **kamano**  No **oyo**  Cannot observe/don’t know **okonyal neno/okia** | | | 1  0  99 |  |  |  |
| C_43 | Does the toilet have the following features?  **Be choo ni nigi gik machal kama?** | Seat **kom**  Handrail **kar luedo**  Steps **steps**  Lighting  **ler** | | | Y/N  Y/N  Y/N  Y/N |  |  |  |
| C_44 | IF TOILET NOT CONNECTED TO SEWER: **KA CHOO OKODUT GI SIWEJ**  Is it possible to empty the pit without breaking the slab?  **Bende inyalo chodho choo ni kaokiketho dierode?** | Yes **kamano**  No **oyo**  Cannot observe/don’t know **okanyal neno/ okia** | | | 1  0  99 |  |  |  |
| C 45 | IF TOILET NOT CONNECTED TO SEWER :  Is the slab stable enough for emptying by Gasia Poa?  **Be choo ni nigi dierot motegno minyalo chodhe gi Gasia Poa?** | Yes **kamano**  No  **Oyoo**  Cannot observe /don’t know **okonyal neno/okia** | | | 1  0  99 |  |  |  |
| C 47 | IF TOILET NOT CONNECTED TO SEWER :  Is the toilet accessible to VTOs (within 50 M of road)? **Be choo ni inyalo chopie ma chodhi gi VTOs ( entiere ma okbor gi mita 50 M kiwuoke e ndara) ?** | Yes **kamano**  No  **Oyoo**  Cannot observe /don’t know **okonyal neno/okia** | | | 1  2  99 |  |  |  |
| *END OF OBSERVATION* ***GIKO MAR NENO*** | | | | | | |  |  |
| C_38 |  |  | | | ___ |  |  |  |
|  |  | *]* | | | ___ |  |  |  |
| C_47 | *CALCULATE SANITATION QUALITY OF TOILET 2* | *If toilet is on the compound , improved, and with 3max households per compartment* | | | HIGH |  |  |  |
|  |  | *Else or no toilet* | | | LOW |  |  |  |
| C_48 | Who paid for the toilet to be constructed? (CAN CIRCLE MULTIPLE)  **Ng’a mane mano chulo pesa mondo oger choo ni?** | Me / my family an/ **jo oda**  Compound neighbors (how many): **Jirande ga ( adi)**_____  Other: **mamoko**:_________________  Don’t know **okia** | | | 1  2  96  99 |  |  |  |
| C_49 | *If* C_48*=1:*  ?Where did you get money to construct the toilet?  **Ni yudo pesa mane I gero go choo kowuok kanye?** | My own savings **pes ga makano**  Borrowed from family/friends **Aolo kowuok ir jooda/ osiepe**  On credit **Gi gowi**  Other: **mamoko** __________________ | | | 1  2  3  4 |  |  |  |
| C_50 | *If* C_48*=1:*  , How long did it take you to finish the payments? **, Nokawi ndalo adi mondo itiek chudo?** | No time - paid immediately **onge saa- nachudo kanyo kanyo**  Under one month  **matine dwe achiel**  1-6 months **1-6 dweche**  Over 6 months  **mang’enyne dweche auchiel**  Other: **mamoko** __________________ | | | 1  2  3  4  5 |  |  |  |
| C_51 | Are there any members of this household that usually don’t use this toilet facility?  **be nitiere jaodi mora mora maok ti gi choo ni?** | Yes **kamano**  No **oyo** | | | 1  0 |  |  |  |
| C_52 | On a scale of 1 – 5, how satisfied are you with this toilet? [USE GRAPHIC SCALE] e wi ratil 1-5,  **iyie marom nade gi choo ni? ( tigi ratil mar graf)** | Very unsatisfied ok **ayiego ahinya**  Somewhat unsatisfied  **ok ayiego matin**  Average **e diere**  Somewhat satisfied **ayiego matin**  Very satisfied **ayiego ahinya**  Don’t know **okia** | | | 1  2  3  4  5  99 | >>C_54 |  |  |
| C_53 | What do you dislike about this toilet? Anything else?  **Ang’o ma okihero kuom choo ni?, gimachielo?**  *(probe as needed; select all that apply)* | Don’t like the type of toilet **okahero aina mar choo ni**?  Dirty **olil**  Bad smell **odung marach**  Health risk **okober gi gima**  Uncomfortable **okolose maber**  Not suitable for children or elderly  **ok ober gi nyithindo kata joma osetii**  risk of falling **inyalo luar**  No privacy **ong’e pandruok**  Inconveniently located  **obor koda**  No water **onge pii**  Security concerns **kwe ma kanyo**  No receptacle for sanitary pads **onge kar keto gige mine (pads)**  Conflict with neighbors  **koko gi jirande**  Pit is full **choo opong**  Pit floods/overflows during rainy season  **choo pong/pukore ndalo koth**  Blockages **odinore**  Other: **mamoko**_______  Don’t know **okia** | | | Y/N  Y/N  Y/N  Y/N  Y/N  Y/N  Y/N  Y/N  Y/N  Y/N  Y/N  Y/N  Y/N  96  99 |  |  |  |
| C_54 | *FOR TENANTS:*  *CALCULATE TENANT CATEGORY* | *If C_18 = HIGH* | | CAT 1 | |  |  |  |
|  |  | *If C_18 = LOW* | | CAT 2 | |  |  |  |
| C_55 | *FOR OWNERS:*  *CALCULATE OWNER CATEGORY* | *If C_02 = 0* | | CAT 5 | |  |  |  |
|  |  | *Else if C_18=LOW or C_25=0 or C_40=LOW* | | CAT 4 | |  |  |  |
|  |  | *Else* | | CAT 3 | |  |  |  |

| **Q No.** | **Remaining toilet questions penjo mag Choo pe modong** | **Options/Answers duoko** |  | **Skip to kadhi nyaka** |
| --- | --- | --- | --- | --- |
| *If we don’t already know what toilet facility the household uses, i.e. if*  *C_01=0 or C_02=0 or (C_22=0 and (C_25=0 or C_44=0))*  Ka okwang’eyo aina mar choo ma ot tiyogo , kaka. Ka *C_01=0 or C_02=0 or (C_22=0 and (C_25=0 or C_44=0))*  *This section should only be about toilets outside the compound*  **E seksoni en mar choope mantiere oko mar dala/plot** | | | | |
| D_01 | What kind of toilet facility do members of your household usually use?  **En aina mane mar choo ma ji ma odni tiyogo?**  [SHOW PICTURES, PROBE, AND READ ALOUD AS NEEDED. IF NOT POSSIBLE TO DETERMINE, ASK PERMISSION TO OBSERVE THE FACILITY]. | **Flush or Pour Flush Toilet to**  **Choo ma iolo pii**  Piped Sewer System  **ma otudi e pipe**  Septic Tank tangi **ma okuny e loo**  Pit Latrine choo **ma okuny matut**  Somewhere Else **kama chielo**  Don't Know Where **okia ni en kanye**  **Pit Latrine choo mokuny matut**  Ventilated Improved Pit Latrine **choo mokuny matut man gi kar muya**  Pit Latrine with Slab  **choo ma okuny matut ma dier ode olos gi simiti**  Pit Latrine with Sitting Toilet  **choo ma okuny to nigi kar bet**  Pit Latrine Without Slab/Open Pit **choo mokuny matut to onge gi dier rot mar simiti**  **Other mamoko**  Composting Toilet (EcoSan)**ekosan**  Container-based Toilet  **choo mar kontaina**  Bucket Toilet **choo mag ndoo**  Hanging Toilet/Hanging Latrine **choo ma olier**  No Facility/Bush/Field  **ji konyore e bungu**  Other: _mamoko______________ | 1  2  3  4  5  6  7  8  9  10  11  12  13  0  96 |  |
| D 02 | Were is this facility located?  **Oge’re gi kanye?** | Less than 30M outside the compound **matine30 M kiwuok oko mar dala/plot**  More than 30M outside the compound **mang’enyne 30 M kiwuok oko mar dala/plot**  Other: _**mamoko**_____  Don’t know **okia** | 1  2  96  99 |  |
| D_03 | How many squat holes (“doors”) does the toilet facility have?  **Gin buche adi miguome ma choo ni nigo?** | _____________ squat holes  **buche miguome** |  |  |
| D_04 | Do you share this toilet facility with other households?  **Be uriwo choo ni gi udi mamoko?** | Yes **kamano**  No oyo  Don’t know okia | 1  0  99 | >>E_00 |
| D05 | Including your own household, how many households use this toilet facility?  **Koriwore gi odi, gin ute adi matiyo gi choo ni?** | Households **ute** | __ |  |
| D 06 | Do you have to pay to use this toilet facility?  **Bende nyaka uchul choo ni?** | Yes  **kamano**  No  **oyoo** | 1  2 |  |
| D_07 | How often do you pay to use this toilet facility  **ichudo ga didi mondo e ka iti gi choo ni?** | Weekly  **wik**  Monthly  **dwe**  Every time I use it  **samora a mora matiyogo**  Don’t know  **okia** | 1  2  3  99 |  |
| D_08 | How much do you spend to use facility (FREQUENCY OF PAYMENT)  **Itiyo gi pesa adi kwom tiyo gi choo ni? ( KAKA ICHUDO)** | A mount in KES:_____  **Pesa e KES_____**  **(**WRITE 99 IF THEY DO NOT KNOW THE AMOINT) ( **ndik 99 ka okia pesa motiyogo)** |  |  |

| **Q No.** | **Emptying services**  ***(And establish Factors D and E)*** | **Options/Answers** | |  | **Skip to** |
| --- | --- | --- | --- | --- | --- |
| E_00 | *CALCULATE NUMBER OF ONSITE SANITATION FACILITIES ON COMPOUND* | *If C_04 = 2-3-6-7-8-9-10 or*  *C_27 = 2-3-6-7-8-9-10* | | Number (N) | >>E_01 |
|  |  | Else | | 0 | >>E_13 |
| *The rest of this section only applies when there is an onsite toilet facility in the compound .*  *Repeat this section N times.****penjo modong e sekson ni bedo man aka kantiere choo e plot. Nuo sekson ni di N*** | | | | | |
| E_01 | When was the last time your toilet was ? Emptied  **Ne en karang’o mane choo ni ochodhi?** | Never been emptied  **pok ne ochodhi**  In the last three months  **dweche adek mokalo**  Between 3 and 12 months ago **kind dweche adek gi apar gariyo mokalo**  Between 1 and 2 years ago kind **higa achiel gi ariyo mokalo**  Over 2 years ago  **moingo higni ariyo mokalo**  **Don’t know okia** | | 0  1  2  3  4  99 | >>E_13  >>E_12 |
| E_02 | What type of service provider conducted the desludging?  **Ne gin jo chodh choo mage mane ochodho ni choo?**  (Show photos of small and large vacuum trucks)  **tang’ne pichni mag gache ma yuayo choo matindo gi madongo** | Large vacuum truck, **Gache madongo** **mayuayo choo,**  Small vacuum truck, **Gache matindo** **mayuayo choo,**  Canter truck with pump  **kanta mangi pamp**  Certified manual emptiers (e.g., Gasia Poa)  **jogo mopuodhi e golo choo ( kaka Gasia-poa**  Illegal manual emptiers jogo **mayuayo choo ma okopuodhi**  Self **An**  Other: **mamoko** ________  Don’t know **okia** | | 1  2  3  4  5  6  96  99 |  |
| E_03 | Who paid for these costs?  (*probe to know whether each category contributed to the cost)*  **Ng’amane mano ochulo pesa no? ( *tem dayo dhoge mondo ing’e nga’no ka ng’ano mano ogolo pesa)*** | Me / my family  **an/ jooda**  Compound neighbors  **jirande ga**  Tenants/ **jopang’o**  Landlord/ **wuon landi**  Caretaker  **jarit landi**  Other: **mamoko**  _________________  Don’t know **okia** | | Y/N  Y/N  Y/N  Y/N  Y/N  99  99 |  |
| E_03d | IF ANSWERED “me” to E 03:  In total, how much did the emptying cost? Including solid waste removal,if any?**Pes duto mane itiyo go e chodho choo ne en pesa adi med nyaka mag golo taka e choo kapo nenitiere ?**  *(Write 0 if none, 99 if don’t know)****dik 0 ka onge, 99 ka okia*** |  | |  |  |
|  |  |  | |  |  |
|  |  |  | |  |  |
|  |  |  | |  |  |
|  |  |  | |  |  |
|  |  | TOTAL COST **PESA TEE** | |  |  |
| E_03e | *IF ANSWERED “me” AND “other tenants” to E_03:*  How many tenants shared these costs?  **Gi jodak adi mane opogore pesa no?** | ______ # of tenants **# namba mar jodak** | |  |  |
| E_ 04 | Did you incur the following additional costs?  **Bende nichulo pesa machielo e gigi?**  ( | Adjacent pit excavation (for birial of waste) **kunyo bur e tie choo( mar yiko minyaga)**  replacing broken slab  **duoko /loso dierot choo mokethi**  fee for authority **pesa jo dito**  **i**  __  **ia** | | Y/N  Y/N  Y/N |  |
| E 05 | *[ If YES at E 04]*  How much did the excavation of an adjacent pit cost?  **[ka en KAMANO E 04]**  **Nokawi pesa adi kunyo bur mantiere e tie choo?** | -----------------KES | |  |  |
| E_ 06 | *[ If YES at E 04]*  How much did repairing the broken slab cost?  **[ka en KAMANO E 04]**  **Nokawi pesa adi chulo pes loso dierot choo mane omuki kikunyo choo?** | ---------KES | |  |  |
| E_07 | *[ If YES at E 04]*  How much was the fee for the authorities?  **[ka en KAMANO E 04]**  **Nokawi pesa adi chulo jodito?** | -----------------KES | |  |  |
| E_08 | *[If YES at E_04]:*  How much was the cost for other costs?  **Ne en pesa adi mag pes mamoko?** | _____________ KES  Don’t know | | 99 |  |
| E_09 | *IF OWNER AND ANSWERED “tenants” to E_ 03:*  Did the tenants then deduct this amount from rent?  **Be ne cho pang’o ongado pesa no e wi pes ot?** | Yes, entire amount  **kamano, pesa no te**  Yes, part of the amount  **kamano, bath pesa no**  No **oyo**  Don’t know **okia** | | 1  2  0  99 |  |
| E_10 | *IF TENANT/CARETAKER AND ANSWERED “me” to E_03:*  Did you then deduct this amount from rent? **Be ni golo pesa no e wi pes ot?** | Yes, entire amount  **kamano, pesa no te**  Yes, part of the amount  **kamano, bath pesa no**  No **oyo**  Don’t know **okia** | | 1  2  0  99 |  |
| E_11 | *FOR TENANTS:*  *CALCULATE TENANT CATEGORY (Factor D)* | *If C_47 = CAT 1* | | | |
|  |  | *If E_00 = NO* | | *Cat 1.0* | *>>F_01* |
|  |  | *Else If E_11 =1* | | *Cat 1.1* |  |
|  |  | *Else* | | *Cat 1.2* |  |
|  |  | *If C_47 = CAT 2* | | | |
|  |  | *If E_00 = NO* | | *Cat 2.0* | *>>F_01* |
|  |  | *If E_11 =1* | | *Cat 2.1* |  |
|  |  | *Else* | | *Cat 2.2* |  |
| E_12 | *FOR OWNERS:*  *CALCULATE OWNER CATEGORY (Factor D)* | *If C_48 = CAT 3* | | |  |
|  |  | *If E_00 = NO* | | *Cat 3.0* | *>>F_01* |
|  |  | *Else* | | *Cat 3.1* |  |
|  |  | *If C_48 = CAT 4* | | | |
|  |  | *If E_00 = NO* | | *Cat 4.0* | *>>F_01* |
|  |  | *Else* | | *Cat 4.1* |  |
| E_13 | *[If E_01 !=0 or 99]:*  On a scale of 1 – 5, how satisfied were you with the emptying service? [USE GRAPHIC SCALE]  **E wi ratil 1-5, ne iyie marom nade e wi choo mane ochodhni? ( tigi ratil mar graf)** | Very unsatisfied **ok ayiego ahinya**  Somewhat unsatisfied  **ok ayiego matin**  Average **e diere**  Somewhat satisfied **ayiego matin**  Very satisfied **ayie ahinya**  Don’t know **okia** | | 1  2  3  4  5  99 |  |
| E_14 | *[If E_01 !=0 or 99]:*  What did you dislike about the emptying service? Anything else?  **Ang’o mane oki ihero kuom chodho ni choo, gimachielo?**  *(probe as needed; select all that apply)* | Nothing, it was okay **onge, nober**  Too costly **neng’o ne tek**  Price changed after first negotiation  **neng’o ne olokore bang winjruok** **mokuongo**  Pit not entirely emptied  **choo nokochodhi te**  Solid waste not removed  **taka nokogol**  Spillages  **luar mar minyaga**  Smell **dung**  Sludge buried onsite  **minyanga okuny e tie choo**  Sludge disposed of in the open **minyaga opuk e pap**  Need to conceal from authorities **pondo ne jo dito**  Other: **mamoko** __________  Don’t know **okia** | | 0  1  2  3  4  5  6  7  8  9  96  99 |  |
| E_15 | *Designate ‘responsible for sanitation emptying.’* | CAT 1.1  CAT 2.1  CAT 3.1  CAT 4.1 | | “RESP” |  |
| E_16 | *FOR OWNERS CAT 3.1 and 4.1 and TENANTS CAT 1.1 and 2.1:*  *UNLESS E_02 = 1 or 2*  What prevented you from using a vacuum truck?  **Ang’o mane omoni tiyo gi jo gari mayuayo minyaga?** | Hasn’t heard about this service **pok a winjo mano**  Doesn’t know how to contact  **Akia kaka anyalo yudo gi**  Accessibility issue **tabu mar chope**  Sludge is too thick **minyaga opoto**  Too costly **neng’o gi tek**  They do not come quickly  **ok gi bi mapiyo**  They do not entirely empty the pit **ok gi chodh choo te?**  Lack of cleanliness **gi koso ler**  Other: mamoko_________  Don’t know **okia** | | 1  2  3  4  5  6  7  8  96  99 |  |
| E_17 | *FOR OWNERS CAT 3.1 and 4.1 and TENANTS CAT 1.1 and 2.1:*  *UNLESS E_02 = 4*  What prevented you from using formal manual emptiers? (Gasia Poa, Vukasasa, Blue Stars)  **Ang’o mane omoni tiyo gi jo chodh choo moyangi? Kaka ( Gasia-poa, Vukasasa, Blue stars)** | Hasn’t heard about this service **pok awinjo mano**  Doesn’t know how to contact  **akia kaka anyalo yudo gi**  Accessibility issue **tabu mar chope**  Too costly **neng’o gi tek**  They do not come quickly  **ok gi bi mapiyo**  They do not entirely empty the pit **ok gi chodh choo te**  Lack of cleanliness **gi koso ler**  Other: **mamoko**_________  Don’t know **okia** | | 1  2  3  4  5  6  96  99 |  |
| E_18 | *FOR OWNERS CAT 3.1 and 4.1 and TENANTS CAT 1.1 and 2.1:*  Have you ever used another type of service provider to eptying the pit? **Benede nisetiyo gi ji mamoko ma chodho choo mondo ochodh ni choo?** | Yes **kamano**  No **oyo**  Don’t know **okia** | |  | >>E_20  >>E_20 |
| E_19 | If so, which type?  **Ka en kamano, ne en aina mane?**  *Select all that apply yier man kare*  *(show photos of small and large vacuum trucks)*  ***(Tang picha mar gache matindo gi madongo mayuayo choo)*** | Large vacuum truck,  **Gache madongo mayuayo choo,**  Small vacuum truck, 8m3  **Gache matindo mayuayo choo**  Canter truck with  **pump kanta mangi pamp**  Certified manual emptiers (e.g., Gasia Poa)  **jo go mopuodhi golo choo( kaka Gasia-poa)**  Illigal manual emptiers jogo **mayuayo choom ok opuodhi**  Self **An**  Other: **mamoko**________  Don’t know **okia** | | 1  2  3  4  5  6  96  99 |  |
| E_20 | On average, how often does your toilet get emptying?  **Kiparo choo ni e chodho bang didi?** | Has been emptied only once or less  **osechodhe dichiel kata matin**  Once per year or more  **dichiel e iga**  Less than once per year  **matine dichiel e iga**  Don’t know **okia** | | 0  1  2  99 | >>E_23  >>E_21  >>E_22  >>E_23 |
| E_21 | Every how many months does your pit get emptied? *(99 if don’t know) wachane didi* ***(99 kaponi okia)* choo ni ichodho bang dweche adi?** | ______________ monthsdwe | |  |  |
| E_22 | Every how many years dies your pit get emptied?  ***(99 if don’t know) wachane didi (99 kaponi okia)*  choo ni ichodho bang igni adi?** | ________________ years iga | |  |  |
| E_23 | *FOR OWNERS CAT 3.1 and 4.1 and TENANTS CAT 1.1 and 2.1 (“RESP”):*  Do you think the toilet will need to get emptied in the next 3 months?  **Iparo ni choo ni ibiro chodhi dueche adek mabiro?** | Yes **kamano**  No **oyo**  Don’t know **okia** | | 1  0  99 | >>E24 |
| E_23b | Do you intend to empty the toilet in the next 3 months?  **Be ichano chodho choo ni e dweche adek mabiro?** |  | |  |  |
| E_24 | TO ENUMERATOR: in your opinion, is there a reasonable need to empty in the next 3 months?  **NE JALO MAPENJO PENJO: ki pachi, bende nitiere aja mar chodhoo e dueche adek mabiro?** | Yes **kamano**  No **oyo**  Don’t know **okia** | | 1  0  99 |  |
| E_25 | *CALCULATE EMPTYING NEED GO KUAND CHOTHO MADUARORE* | *Based on answers to questions E_01, E_23, E_24 koluwore gi duoko E_01,E_23,E_24* | | YES kamano |  |
|  |  |  | | NO oyo |  |
| E_26 | Is the *slab stable enough for emptying by Gasia Poa*   **Be choo ni nigi dierotmotegno minyalom chod gi Gasia Poa?** | Yes **kamano**  No **oyo**  Cannot observe/don’t know **okanyal nene/ okia** | | 1  0  99 |  |
| E_27 | Is the toilet accessible toVTOs?(within 50M of road)?  ***Be choo ni inyalo chopie ma chodh gi VTO’s ( entiere ma okbor gi mita 50 kowuok e ndara)?*** | Yes **kamano**  No **oyo**  Cannot observe/don’t know **okanyal nene/ okia** | | 1  0  99 |  |
| E_28 | *If (E_12=1.1 or 2.1) or (E_13=3.1 or 4.1)*  *CALCULATE FACTOR E*  *GO KWANO MAR E* | If E_25=YES | | | |
|  |  | If E_26=1 and E_27=1 | A-both | |  |
|  |  | If E_26=1 and E_27=0 | A-GP | |  |
|  |  | If E_26=0 and E_27=1 | A-VTO | |  |
|  |  | If E_26=0 and E_27=0 | B | |  |
|  |  | Else | B | |  |
| **REPEAT QUESTIONS E_01 to E_28 for second onsite toilet, if any Nuo penjo E_01 nyaka E_28 ne choo mar udi marariyo, ka po ni nitie** | | | | | |

| **Q No.** | **Final Categories**  **Okang’ mogik** | **Options/Answers**  **Dwoko** |  | **Skip to**  **Chikri nyaka** |
| --- | --- | --- | --- | --- |
| F_01 | *FOR TENANTS:*  *CALCULATE FINAL CATEGORY*  *NE JI MA CHULO UDI:*  *GWO KWANO MAR OKANG’ MOGIK* | Concatenate [E_12, “-“, E_28] | |  |
| F_02 | *FOR OWNERS:*  *CALCULATE FINAL CATEGORY*  *NE WEG UDI:*  ***GO KWANO MAR OKANG’ MOGIK*** | Concatenate [E_13, “-“, E_28] | |  |

|  |  |  |  |  |
| --- | --- | --- | --- | --- |
|  |  |  |  |  |
|  |  |  |  |  |
|  |  |  |  |  |
|  |  |  | 9 |  |
|  |  |  |  |  |
|  |  |  |  |  |

| **Q No.** | **TENANTS (CAT 2) WEG UDI(CAT2)**  **WTP for access to sanitation option**  **WTP mar yuto mar yore mag ler** | **Options/Answers**  **Dwoko** |  | **Skip to**  **Chikri nyaka** |
| --- | --- | --- | --- | --- |
| H_01 | Now we would like to show you some different sanitation options.  Koro wanyalo hero mar tang’oni yore ma opogore mag rito ler.  READ DESCRIPTION AND SHOW PHOTOS OF SELECTED sanitation options.  SOM NDIKO KASTO ITANG’ PICHNI MA OYIER  ASK THE FOLLOWING FOR EACH OPTION PRESENTED.  **PENJI PENJO MACHAL KAMAE NE YIERO MA OTANG’** | | | |
|  | *Have you read the script and shown the graphics to the respondent?*  ***Be isesomo ndiko kod pichni ne jaduok penjo?*** | Yes **Kamano**  No **Oyo** | 1  0 |  |
| H_02 | Imagine this sanitation option was available in your rented home.  **Par ane ni rito ler machal kamae nitie e odi ma ikodesa ni.**    Would you be willing to pay an additional X/month (randomized) on your rent?  **Inyalo yie chulo pesa ewi ma ichuloga e giko dwe kuom ot ma ikodesa?** | Yes **Kamano**  No **Oyo**  Don’t know **Okia** | 1  0  99 |  |
| H_03 | *[Y amount to be adjusted up or down based on H_02]*  ***[ pesa ma nyalo medore kata dok chien kaluwore kod H_02]***  Would you be willing to pay an additional Y/month on your rent?  **Inyalo yie medo pesa marom gi Y e dwe kwa dwe e pesa ma ichulo mar ot?** | Yes **Kamano**  No **Oyo**  Don’t know **Okia** | 1  0  99 |  |
| H_04 | What is the highest additional amount you would be willing to pay on your rent for this level of service to be present in your rented home?  **Omuom mang’eny marom nade ma inyalo yie medo e dwe e ot ma ikodesa ka luwore kod chenro ma imiyi?** | Amount:________________  **Pesa**:________________ |  |  |

| **Q No.** | **TENANTS (CAT 1.1, 1.2, 2.1, 2.2)**  **JII MA OKODESA OT(CAT 1.1,1.2,2.2)**  **WTP for emptying service**  **WTP mar chodho minyaga** | **Options/Answers**  **Dwoko** |  | **Skip to**  **Chikri nyaka** |
| --- | --- | --- | --- | --- |
| I_01 | Now we would like to show you different types of emptying services.  **Koro wadwaro tang’onu yore ma opogore mag chodho chope.**  READ DESCRIPTION AND SHOW PHOTOS OF SELECTED emptying services.  **SOM NDIKO KASTO ITANG’ PICHNI MAR YORE MAG CHODHO CHOPE MA OYIER**  **ASK THE FOLLOWING FOR EACH OPTION PRESENTED.**  **PENJ PENJOGI NE YIERO MA OMIYI** | | | |
|  | *Have you read the script and shown the graphics to the respondent?*  ***Be isesomo ndiko ma itang’o pichni ne joduok penjo?*** | Yes **Kamano**  No **Oyo** | 1  0 |  |
| I_02 | Imagine this emptying service was available for the toilet facility on this property.  **Par ane ni chenro mar chodho choo ne nitie e plodi ni.**  Would you be willing to pay an additional X/month (randomized) on your rent?  **Be inyalo yie chulo pesa mageny maromo gi X- e dwe ka dwe mar ot?** | Yes **Kamano**  No **Oyo**  Don’t know **Okia** | 1  0  99 |  |
| I_03 | *[Y amount to be adjusted up or down based on I_02]*  ***Y Pesa minyalo medi kata duoko piny kaluore gi I_02***  Would you be willing to pay an additional Y/month on your rent?  **Inyalo yie medo pesa marom gi Y e dwe ka dwe e pesa ma ichulo mar ot?** | Yes **kamano**  No **oyo**  Don’t know **okia** | 1  0  99 |  |
| I_04 | What is the highest additional amount you would be willing to pay on your rent for this level of service to be present in your rented home?  **Omuom mang’eny marom nade ma inyalo yie medo e dwe e ot ma ikodesa ka luwore kod chenro ma imiyi?** | Amount:_**pesa:**_______________ |  |  |

| **Q No.** | **OWNERS (CAT 4-5) WEG GI (CAT 4-5)**  **WTP for toilet construction**  **WTP Mar gero choo** | **Options/Answers duoko** |  | **Skip to chikri nyaka** |
| --- | --- | --- | --- | --- |
| J_01 | Now we would like to show you different sanitation options.  READ DESCRIPTION AND SHOW PHOTOS OF SELECTED SANITATION OPTIONS.  ASK THE FOLLOWING FOR EACH OPTION PRESENTED.  **Koro wadwa tang’o ni yore mopogore opogore mar rito ler:**  **SOM NDIKO KASTO ITANG’ PICHNI MA OYIER MAG RITO LER. PENJ PENJO GI NE YIERO MOPOGORE MOTANG’NI** | | | |
|  | *Have you read the script and shown the graphics to the respondent?*  ***Be isesomo ndiko ma otang’ gi pichni ne jaduok penjo?*** | Yes **Kamano**  No **Oyo** | 1  0 |  |
| J_02 | Suppose this sanitation facility was available for you to construct for a total a mount of X (randomized) ( including materials, transport and labor .  Would you be willing to pay X ?  **Kadebedi ni yiero mar rito ler ni nyalo yudo mar gero choo mar pesa X. ( ma oriwo matiriol, ting’o kod jotich) Be inyaloyie chulo pesa maromo(X)?** | Yes **Kamano**  No **Oyo**  Don’t know **Okia** | 1  0  99 |  |
| J_03 | *[Y amount to be adjusted up or down based on J_02]*  ***Y Pesa minyalo medi kata duoko piny kaluore gi j_02***  Imagine this sanitation option was available to you for Y amount.  Would you be willing to pay Y ?  **Iparo ni yiero mar rito ler ni ka otimni ne y. Be inyaloyie chulo pesa maromo(y)?** | Yes **Kamano**  No **Oyo**  Don’t know **Okia** | 1  0  99 |  |
| J_04 | What is the maximum to you would be willing to pay for this sanitation solution?  **Omuom marom nade mang’eny mogik ma inyalo yie chulo ne yoo mar rito ler ni?** | Amount:_**Pesa**:_______________ |  |  |
| J_05 | Suppose there is an option to purchase for this sanitation facility within 12 months payments of x (randomized ) amount  Would you be willing to pay X (randomized) per month?  **Kadebedi nia nitie yiero mar chudo ne yor rito ler matin matin kuom dweche apar gi ariyo. Be inyaloyie chulo X e dwe ka dwe?** | Yes **Kamano**  No **Oyo**  Don’t know **Okia** | 1  0  99 |  |
| J_06 | *[Y amount to be adjusted up or down based on J_05]*  ***Y Pesa minyalo medi kata duoko piny kaluore gi j_05***  Would you be willing to pay Y per month?  **Be inyalo yie chulo pesa maromo Y dwe ka dwe?** | Yes **Kamano**  No **Oyo**  Don’t know **Okia** | 1  0  99 |  |
| J_07 | What is the maximum you would be willing to pay to construct this sanitation facility (monthly over 12 months)?  **En pesa adi mangeny’ minyalo yie chulo mar gero choo dwe ka dwe makalo dweche 12 ?** | Amount:_**Pesa:**_______________ |  |  |
| J_08 | If you constructed this sanitation option, would you change the rent for your tenants?  **Ka ne igero yor letr ni, binyalo loko pesa ute ma dwe ka dwe?** | Yes **Kamano**  No **Oyo**  Don’t know **Okia** | 1  0  99 | >>  >> |
| J_09 | How much would you increase rent per month for your tenants?  **Inyalo medo pesa adi mar uteni dwe ka dwe?** | Amount:_**Pesa:**_______________ |  |  |

| **Q No.** | **OWNERS (CAT 1.1, 2.1, 3.1, 4.1) WEG GI(CAT 1.1,2.1,3.1,4.1)**  **WTP for emptying service** | **Options/Answers**  **Dwoko** |  | **Skip to**  **Chikri nyaka** |
| --- | --- | --- | --- | --- |
| K_01 | Now we would like to show you some different emptying service options.  READ DESCRIPTION AND SHOW PHOTOS OF SELECTED EMPTYING SERVICES.  ASK THE FOLLOWING FOR EACH OPTION PRESENTED.  **Koro wadwaro tang’oni yore mopogore mag chodho choo.**  **SOM NDIKO KASTO ITANG PICHNI MAG YORE MAG CHODHO CHOO.**  **PENJI PENJOGI NE YIERO MORO AMORA MA OYIER.** | | | |
|  | *Have you read the script and shown the graphics to the respondent?*  ***Be isesomo ndiko ma otang’ gi pichni ne jaduok penjo?*** | Yes **Kamano**  No **Oyo** | 1  0 |  |
| K_02 | Suppose this emptying service was available to you for X (randomized) amount.  Would you be willing to pay X ?  **Kadebedi ni yiero mar chodho choo ni ka ne ntiere ni mar pesa X . Be inyalo yie chulo pesa X?** | Yes **Kamano**  No **Oyo**  Don’t know **Okia** | 1  0  99 |  |
| K_03 | *[Y amount to be adjusted up or down based on K_02]*  ***[Pesa Y medore kata dok chien kalowere gi K_0***  Would you be willing to pay Y ?  **Be inyalo yie chulo pesa Y?** | Yes **Kamano**  No **Oyo**  Don’t know **Okia** | 1  0  99 |  |
| K_04 | What is the maximum a mount you would be willing to pay for this emptying service?  **En pesa adi mangeny’ minyaloyie chulo dwe ka dwe e yor chodho choo?** | Amount: **Pesa:**________________ |  |  |
| K_05 | Suppose there is an option for you to pay the Kisumu WWWAa monthly fee that makes you eligible to request 1 emptying service per year at any time you want it.  Would you be willing to pay X (randomized) per month?  **Kadebedi ni nitie yiero mar chulo Kisumu WWWA dwe ka dwe ma koro miyo inyalo kwayo mondo ochodhni choo ni samoro amora ma idwaro e higa. Be ibiroyie chulo pesa X dwe ka dwe?** | Yes **Kamano**  No **Oyo**  Don’t know **Okia** | 1  0  99 |  |
| K_06 | *[Y amount to be adjusted up or down based on K_05]*  *[Pesa Y medore kata dok chien kalowere gi K_05]*  Would you be willing to pay Y per month?  **Be inyalo yie chulo pesa Y dwe ka dwe?** | Yes **Kamano**  No **Oyo**  Don’t know **Okia** | 1  0  99 |  |
| K_07 | What is the maximum amount you would be willing to pay per month for this emptying subscription?  **En pesa adi mangeny’ minyalo yie chulo dwe ka dwe e yor chodho choo ni ?** | Amount:_**Pesa:**_______________ |  |  |
| K_08 | If you used this emptying service, would you add to your rent per month for your tenants?  **Ka itiyo gi yiero ni, binyalo medo pesa ute ma dwe ka dwe?** | Yes **kamano**  No **oyo**  Don’t know **okia** | 1  0  99 | >>  >> |
| K_09 | How much would you increase rent per month for your tenants?  **Inyalo medo pesa adi mar uteni dwe ka dwe?** | Amount:_**pesa** _______________ |  |  |

| **Q No.** | **Water** | **Options/Answers** |  | **Skip to** |
| --- | --- | --- | --- | --- |
| P_01 | [*If Cat 2, 4, or 5*]  Which of the three sanitation options would you prefer?  **E kind yore mag ler a dek gi ere miyalo yiero?** | Pour-flush to lined pit **choo miole pii kadhi e bugo moger kawuok piny**  Pour-flush to sewer  **choo miole pii to otudi e siwej**  Container-based sanitation  **choo mar kontaina**  None  **onge**  Don't know  **okia** | 1  2  3  0  99 |  |
| P_02 | Which emptying service do you prefer?  **Aina mane mar chodho choo madiher?** | VTOs  **VTO’s**  Gasia Posa  **Gasia Poa**  Other manual (illegal) emptiers  **jo choth choo mokopuodhi**  Don't know  **okia** | 1  2  3  99 |  |
| P_03 | How would you prefer to pay for a new toilet?  **Diher aina mane mar chulo choo?** | Lump sum payment (all at once) **chudo dichiel**  In installments  **chudo matin matin**  Combination of some up front and the rest in installments  **chudo mageny mokuong’o kasto modong ichulo mos mos**  Other: __**mamoko**_____________  Don't know  **okia** | 1  2  3  96  99 |  |
| P_04 | How would you prefer to pay for emptying services?  **Diher aina mane mar chulo chodho choo?** | Lump sum payment (all at once), at time of emptying  **chudo dichiel seche michodho choo**  As a subscription  **kaka matin matin**  Combination of some in lump sum at time of emptying and some subscription payment  **kiriwo chudo dichiel seche michodho choo to gi matin matin**  Other: __**mamoko**_____________  Don't know  **okia** | 1  2  3  96  99 |  |
| **Q No.** | **Water** | **Options/Answers** |  | **Skip to** |
| L_01 | Is there a piped water connection on the premises of this compound?  **Bentiere pii motudi mar pipe e ute gi ?** | Yes **kamano**  No **oyo**  Don’t know **okia** | 1  0  99 |  |
| L_02 | What is the main source of water for your household’s activities such as cooking and handwashing?  Pii ma odni tiyo go ( tedo gi luoko) wuok kanye?  [SELECT ONE. PROBE AS NEEDED. IF HOUSEHOLD STATES WATER VENDOR, PROBE ON WATER SOURCE]. | **Piped Water Pii maringo e paip**  Piped Into Dwelling  **Paip madonjo e ute ma jii odakie**  Piped To Yard/Plot  **Paip ma donjo e plot**  Piped To Neighbor  **Paip madhi ne jirani**  Public Tap/Kiosk  **Pii ma iuso e fereji e kiosk**  **Other Mamoko**  Tube Well or Borehole  **Kisima**  Dug Well  **Yao**  Protected Well  **Yao ma nigi ohinga**  Unprotected Well  **Yao ma oyaore**  Protected Spring  **Achia ma oriti**  Unprotected Spring  **Achia ma ok oriti**  Rainwater harvesting  **Pii koth ma oya e trado**  Tanker Truck  **Pii lori**  Cart With Small Tank  **Pii ma oting’ e Mkokoteni**  Surface Water **Oula**  (River/Dam/Lake/Pond/ Stream/Canal/Irrigation Channel)  **Aora/Yao/Nam/Aora/Irigason**  Bottled Water **Pii chupa**  Other: _______________  **Mamoko**  Don’t know **Okia** | 1  2  3  4  5  6  7  8  9  10  11  12  13  14  15  96  99 |  |
| L_03 | *ONLY ASK IF WATER SOURCE IS* ***NOT****:*   - *PIPED INTO DWELLING* - *PIPED TO YARD/PLOT*   *OTHERWISE, SKIP TO L_04*  ***PENJI MANA KA KAMA PII WUOKE OK OTUDI PAIP NYAKA E UTE JII KATA NYAKA E PLOT.KA OK KAMANO TO CHIKRI NYAKA L_04***  Where is that water source located?  **Pii no yudore kanye?** | In Own Dwelling  **E ot ma idake**  In Own Yard/Plot  **E plot ni**  Elsewhere **Kamachielo**  Other: _____**Mamoko_**_________  Don’t know  **Okia** | 1  2  3  96  99 | >>L_05  >>L_05 |
| L_04 | How long does it take to go there, get water, and come back?  **Kawo dakika marom nade mondo, iom pii miduogi?** | Minutes:  **dakika** |  |  |
|  |  | Don’t know **okia** | 99 |  |
| L_05 |  |  |  |  |
|  |  |  |  |  |
|  |  |  |  |  |
|  |  |  |  |  |
|  |  |  |  |  |

| **Q No.** | **Household Finances and Assets** | **Options/Answers** |  | **Skip to** |
| --- | --- | --- | --- | --- |
| M_01 | What is your household TOTAL income per month?  **Yuto mar odni dwe ka dwe en pesa adi?**  [PROBLE FOR ALL INCOME SOURCES. READ RANGE.]  [**DAA DHOGE NE YORE TEE MAG YUTO]** | <3,000  3,000 – <5,000  5,000 – <7,000  7,000 – <10,000  10,000 – <23,000  23,000 – <50,000  50,000 – <120,00  8≥120,000  Don’t know Okia  Refuse to answer Otamore dwoko | 1  2  3  4  5  6  7  8  9  10  11  99  98 |  |
| M_03 | Does your household have …?  **Be odi nigi?** | Electricity **Sitima** Y / N / DK  Radio **Nyakalondo** Y / N / DK  Television **Wang’ liech** Y / N / DK  Non-mobile phone/landline **Ong’we yamo** **mar tol** Y / N / DK  Refrigerator **frig** Y / N / DK  Solar panel **Sola** Y / N / DK  Table **Mesa** Y / N / DK  Chair **Kom** Y / N / DK  Sofa **Sofa** Y / N / DK  Bed **Kitanda** Y / N / DK  Cupboard **Kabat** Y / N / DK  Wall clock **Saa korot** Y / N / DK  Microwave oven **maikrowev** Y / N / DK  DVD player **Thumb DVD** Y / N / DK  Cassette or CD player **Thumb Kaset** Y / N / DK | |  |
| M_04 | Does any member of this household own a..?  **Be ng’at a ng’ata ma ntie e odni nigi..?** | Watch **Saa** Y / N / DK  Mobile phone **Ong’we yamo** Y / N / DK  Bicycle **Ndiga** Y / N / DK  Motorcycle/motor scooter **Apiko** Y / N / DK  Animal-drawn cart  **Mkokoteni** Y / N / DK  Car or truck **gari/lori** Y / N / DK  Boat with a motor **Yie andururu** Y / N / DK | |  |
| M_05 | What type of fuel does your household primarily use for cooking?  **Odni tiyo gi ang’a ahinya e yor tedo?** | Electricity **Sitima**  LPG/Natural gas **Gas**  Biogas **Bayogas**  Paraffin / Kerosene **Mauta/mafuta**  Coal, lignite**Kol**  Charcoal **Makaa**  Wood **Yien**  Straw/shrubs/grass **Lum**  Agricultural crop**Tiang**’  Animal dung **Owuoyo**  No food cooked in household **Chiemo ok tedi e odno**  Other: _______________**Mamoko**  Don’t know **Okia** | 1  2  3  4  5  6  7  8  9  10  0  96  99 |  |
| M_06 | How many rooms does this household occupy for living, eating and sleeping ?*do not count bathroom, kitchen or toilet*  **Odni nigi room adi , kuan kor nindo, kor budho to gi kar chiemo ? kik ikuan baf, jikon to gi choo** | Number of rooms:  **Kuonde nindo**: |  |  |
| M_07 | In what type of housing does your household live? [OBSERVE OR ASK]  **Jo odi odak e ot aina mane?[NG’I KATA PENJI]** | House (single family), own compound, or bungalow  **dala mar famili achiel**  Bedsitter **bet sita**  Flat / Apartment **gorofa**  Servants Quarters **od jatich**  County houses, municipal houses **ute sirikal mar kaunti**  Shack, container, tent (Improvised home)**ute kontainer**  Living quarters attached to shop/workplace  **oong’e ott odakie kama otiye**  Othe r  **mamoko:** _______________ | 1  2  3  4  5  7  8  96 |  |
| M_08 | Main material of the floor of respondent’s home  **matiriol molos go dierod jaduok penjo** | Earth/sand **loo/** **kuoyo**  Dung **owuoyo**  Wood planks **bape**  Palm/Bamboo **bambu**  Parquet or Polished Wood  **bape mopang lare mar dierot**  Vinyl or Asphalt Strips strips  **bape mopa maler kaka tael**  Ceramic Tiles **tael**  Cement **simiti**  Carpet **kapet**  Other: _**mamoko**______________  Don’t know **okia** | 1  2  3  4  5  6  7  8  9  96  99 |  |
| M_09 | Main material of the roof of respondent’s home  Matiriol molos godo wi ot jaduok penjo | No Roof onge wi tado  Thatch/Grass/Makuti lum  Dung/Mud/Sod thuodho  Iron Sheets mabati  Tin Cans kans mag tin  Asbestos Sheet mabat asbesto  Concrete morogi  Tiles tael  Other: mamoko_______________  Don’t know okia | 0  1  2  3  4  5  6  7  96  99 |  |
| M_10 | Main material of the walls of respondent’s home  *Covers more than 50% of the wall*  **Matiriol molos godo kor ot jaduok penjo (*mokao atama 50)*** | No Walls **onge korot**  Cane/Palm/Trunks **odundu**  Dung/Mud/Sod **owuoyo/loo**  Bamboo with Mud  **bambu moriw gi loo**  Stone with mud **kidi moriw gi loo**  Uncovered adobe (mud bricks) **matafare mok oum**  Plywood **bape mayom**  Cardboard **bape matek**  Reused wood **yiende moti**  Iron sheets **mabati**  Cement **simiti**  Stone with Lime/Cement  **kite gi lime**  Bricks (kiln dried)  **matafare mowang**  Cement Blocks **blok mar simiti**  Covered Adobe (plastered mud bricks)  **matafare gi loo mogo simiti**  Wood planks/shingles  **yiende mopangi**  Other:mamoko _______________  Don’t know **okia** | 0  1  2  3  4  5  6  7  8  9  10  11  12  13  14  15  96  99 |  |
| M_11 | Does your household use M-Pesa?  **Be odi ka itiye gi M-Pesa?** | Yes **kamano**  No **oyo**  Don’t know  **okia** | 1  0  99 |  |

| **Q No.** | **CAT 1.1-A, 2.1-A, 3.1-A, 4.1-A**  **Emptying vouchers** | **Options/Answers** |  | **Skip to** |
| --- | --- | --- | --- | --- |
| N_01 | You have been randomly selected to receive a voucher for emptying services. We will be giving you this voucher, which is like a coupon, to allow you to get an emptying job at a cheaper price. <Demonstrate with voucher.> On this voucher, you can see what type of emptying service you are eligible to buy. Should you choose to get this service, you can see the price you’d be required to pay (no more, no less). You can also see the price that the emptying service normally costs. This voucher is only good until X date, 2019 and you are only able to redeem it with supplier______. This supplier is available every day from x-y___ and can be contacted through this number. Please remember that you are not required to buy the service.  **In achiel kuom jogo moyier mondo oyud oboke mar chudo mar chodho choo. Wabiro miyi oboke mar chudo ni, machal kaka gima osechul , ma biro konyi e yo chodho choo e neng’o moyueyo. < pimne gi oboke mar chudo>. E oboke ni mar chudo, inyalo neno aina mar chotho ma oyieni giewo. Kadi po ni iyie kode, inyalo neno pesa mibiro duar ni ichul ( onge mangen’y kata matin). Inyalo neno neng’o mar chodho choo mapile. Oboke mar chudo ni tiyo nyaka tarik X ,2019 kendo inyalo ti kode gi jalno ma miyi. Jalno ma miyi gi biro yudore koya tarik X-Y kendo onyaloyudore ka okalo e namba ni. Par ni ok onego ichul/inyiew chenro ni.** | | | |
|  | *Have you read the script and shown the voucher to the respondent?*  ***Be isomo diko no mitango tipo mar oboke mar chudo ne jaduok penjo?*** | Yes **kamano**  No **oyo** | 1  0 |  |
| N_02 | *Which voucher type did the respondent receive?*  ***En opoke mane mar chudo ma jaduok penjo oyudo?*** | Service type:  **mane**  Subsidy amount:  **Pesa mong’adne:** |  |  |
| N_03 | *What is the voucher number on the voucher they received?*  ***Namba mar oboke mar chudo magiyudo ni en mane?*** | __ __ __ ___ |  |  |
| N_04 | *Take a photo of the voucher*  ***kaw pich oboke mar chudo ni*** |  |  |  |

| **Q No.** | **CAT 4-5**  **Toilet vouchers**  **oboke mar chudo mar choo** | **Options/Answers**  **duoko** |  | **Skip to**  **Kadhi nyaka** |
| --- | --- | --- | --- | --- |
| O_01 | You have been randomly selected to receive a voucher for constructing a toilet. We will be giving you this voucher, which is like a coupon, to allow you to buy a sanitation solution at a cheaper price. <Demonstrate with voucher.> On this voucher, you can see what type of sanitation solution you are eligible to buy. Should you choose to buy the solution, you can see the price you’d be required to pay (no more, no less). You can also see the price that the sanitation solution normally costs. This voucher is only good until X date, 2019 and you are only able to redeem it at ____storE______. This store is located ______ and is open ___everyday from x-y___. Please remember that you are not required to buy the sanitation option.    **In achiel kuom jogo moyier mondo oyud oboke mar chudo mar gero choo. Wabiro miyi oboke mar chudo ni, machal kaka gima osechul , ma biro konyi e e ng’iewo yore ler e neng’o moyueyo. < pimne gi oboke mar chudo>. E oboke ni mar chudo, inyalo neno aina mag yore mag ler ma oyieni giewo. Kadi po ni iyie kode, inyalo neno pesa mibiro duar ni ichul ( onge mangen’y kata matin). Inyalo neno neng’o mar yore mag ler mapile. Oboke mar chudo ni tiyo nyaka tarik X ,2019 kendo inyalo ti kode -----------e duka -------------. Duka ni nitiere -----------kendo oyawe ---------------pile ka pile koya tarik X-Y. Par ni ok onego ichul/inyiew chenro ni.** | | | |
|  | *Have you read the script and shown the voucher to the respondent?*  ***Be isomo diko no mitango tipo mar oboke mar chudo ne jaduok penjo?*** | Yes **kamano**  No **oyo** | 1  0 |  |
| O_02 | *Which voucher type did the respondent receive?*  ***En opoke mane mar chudo ma jaduok penjo oyudo?*** | Service type:  **Mane:**  Subsidy amount:  **Pesa mong’adne:** |  |  |
| O_03 | *What is the voucher number on the voucher they received?*  ***Namba mar oboke mar chudo magiyudo ni en mane?*** | __ __ __ ___ |  |  |
| O_04 | *Take a photo of the voucher*  ***kaw pich oboke mar chudo ni*** |  |  |  |
|  |  |  |  |  |
|  |  | ____________________ |  |  |

| Text | **This concludes the survey. Is there anything else you’d like us to know?**  Mano e giko mar nonro ni. Be nitiere gimora a mora migombo ni wang’e?  The interview is finished. Thank you very much! **Penjo orumo. Erokamano ahinya!** | Comments: |
| --- | --- | --- |
